# Strategies to Improve the Thermoelectric Figure of Merit in Thermoelectric Functional Materials

**DOI:** 10.3389/fchem.2022.865281

**Published:** 2022-05-19

**Authors:** Yan Sun, Yue Liu, Ruichuan Li, Yanshuai Li, Shizheng Bai

**Affiliations:** ^1^ College of Material Science Engineer, Liaoning Technical University, Fuxin, China; ^2^ College of Material Science Engineer, Harbin Institute of Technology, Harbin, China

**Keywords:** thermoelectric materials, *ZT* value, seebeck coefficient, electrical conductivity, thermal conductivity

## Abstract

In recent years, thermoelectric functional materials have been widely concerned in temperature difference power generation, electric refrigeration and integrated circui, and so on. In this paper, the design and research progress of thermoelectric materials around lifting *ZT* value in recent years are reviewed. Optimizing the carrier concentration to improve the Seebeck coefficient, the steady improvement of carrier mobility and the influence of energy band engineering on thermoelectric performance are discussed. In addition, the impact of lattice thermal conductivity on *ZT* value is also significant. We discuss the general law that the synergistic effect of different dimensions, scales, and crystal structures can reduce lattice thermal conductivity, and introduce the new application of electro-acoustic decoupling in thermoelectric materials. Finally, the research of thermoelectric materials is summarized and prospected in the hope of providing practical ideas for expanding the application and scale industrialization of thermoelectric devices.

## Introduction

**FIGURE 1:**
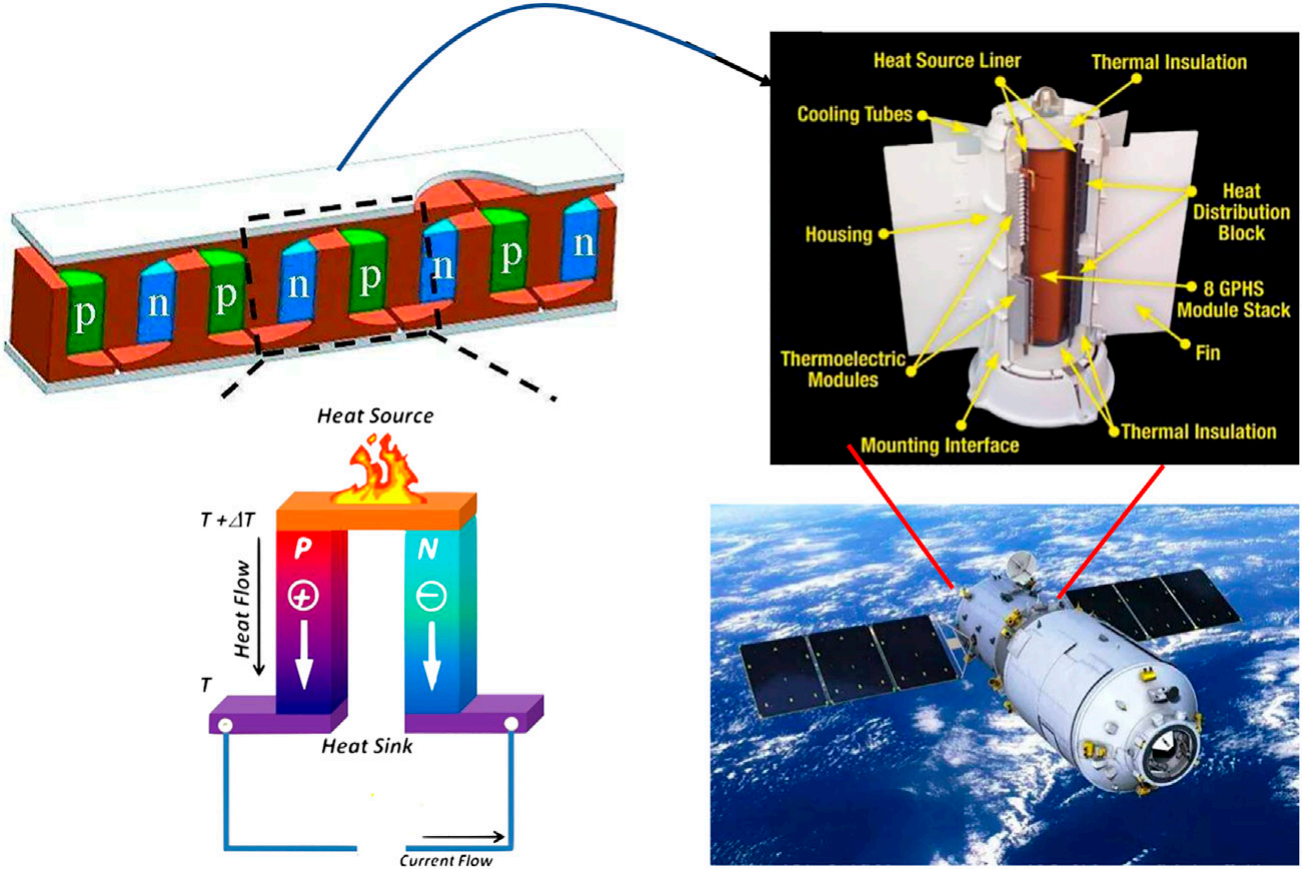
Application and working mechanism of thermoelectric materials in aerospace.

With the progress of society, energy and environmental issues have become the most severe challenges for humanity in the new era. Especially for fossil energy, the large amount of waste heat generated in the combustion process cannot be reused, and the energy utilization rate is meager (Vining, 2009). To solve this problem, the research of a thermoelectric functional material that directly converts heat energy and electric energy to each other has received extensive attention. With their small size, high reliability, long life, high temperature sensitivity, and environmental friendliness, thermoelectric materials (TE) have become a current research hotspot (Figure 1). It is now mainly used in crucial new energy directions such as space probes, industrial waste heat utilization, and solar composite power generation (Poudel et al., 2008; Suarez et al., 2016). Since the middle of the last century, the performance of TE materials has been dramatically improved, which is the key to improving the energy conversion efficiency of TE devices. Solid-state TE devices can not only use the Seebeck effect to convert heat into electricity but also the Peltier effect to provide cooling.

The energy conversion efficiency of TE material power generation equipment is defined as the output electric energy (P) divided by the provided thermal energy (Q) (Yang and Caillat, 2006):

$$\eta =\frac{P}{Q}=\frac{\Delta T}{T_{H}}\left(\frac{\sqrt{1+\bar{ZT}}-1}{\sqrt{1+\bar{ZT}}+\frac{T_{C}}{T_{H}}}\right)$$ (1)

**FIGURE 2:**
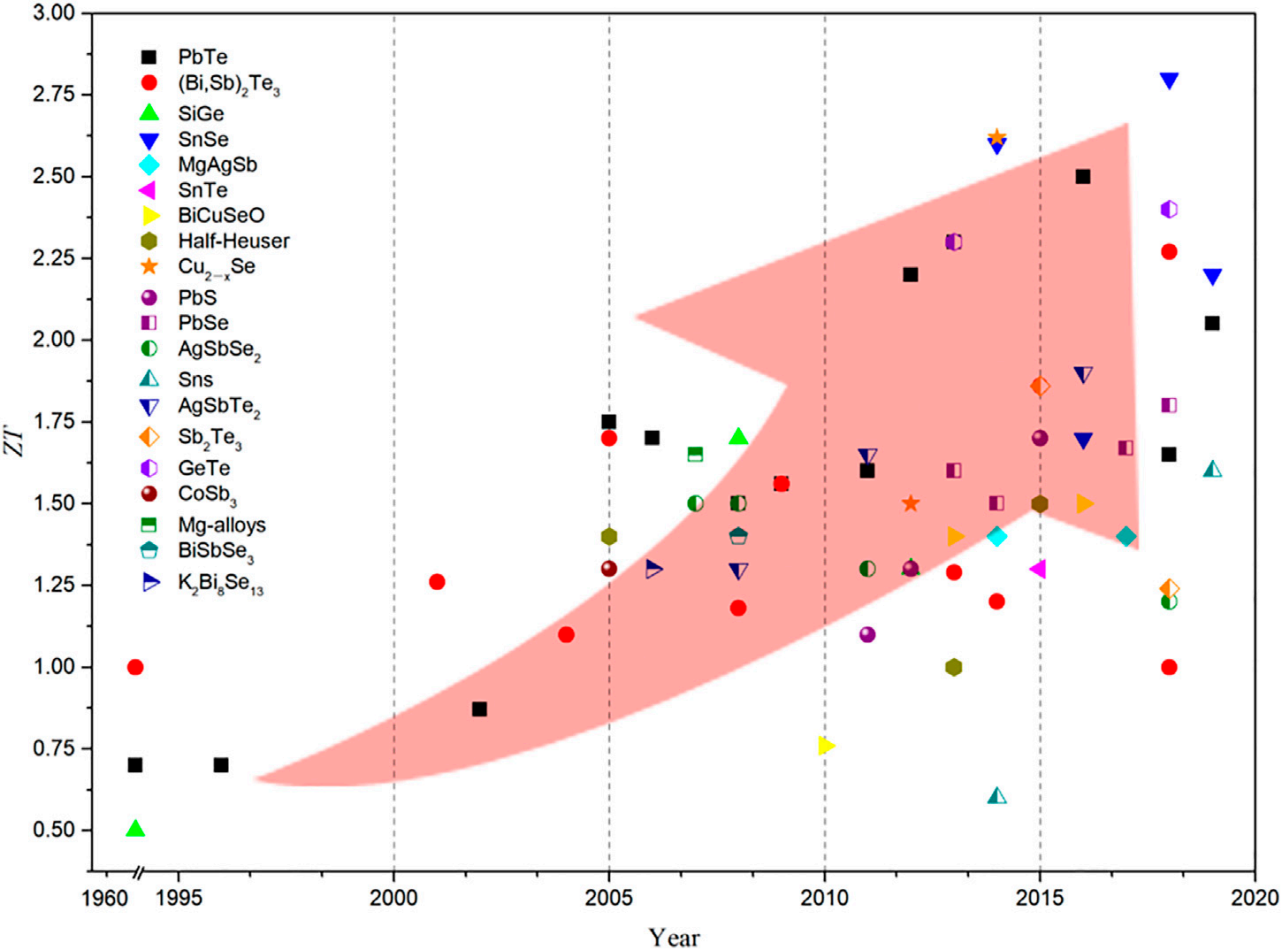
The figure of merit of TE, thermoelectricity in recent years (Ioffe et al., 1959; Cutler et al., 1964; Hicks and Dresselhaus, 1993; Horbach et al., 2001; Yang et al., 2001; Dashevsky et al., 2002; Kuznetsov et al., 2002; Hsu et al., 2004; Shutoh and Sakurada, 2005; Heremans et al., 2008; Poudel et al., 2008; Wang et al., 2008; Yang et al., 2008; Zhao et al., 2008; Xie et al., 2009; Bergum et al., 2011; Du et al., 2011; Levin et al., 2011; Pei et al., 2011; Li et al., 2012; Liu et al., 2012; Chen et al., 2013; Gelbstein et al., 2013; Sui et al., 2013; Yamini et al., 2013; Yan et al., 2013; Hu et al., 2014; Lee et al., 2014; Tan et al., 2014; Wu et al., 2014; Zhao et al., 2014a; Zhao et al., 2014b; Zhong et al., 2014; Kim et al., 2015; Kraemer et al., 2015; Tan et al., 2015a; Tang et al., 2015; Liu et al., 2016; Tan et al., 2016a; Liu et al., 2017; Roychowdhury et al., 2017; Zhang et al., 2017; Chang et al., 2018; Li et al., 2018; Zhou et al., 2018; Xiang et al., 2019; Chong et al., 2020; Feng et al., 2020; Qin et al., 2020; Yvenou et al., 2020; Selimefendigil et al., 2021; Su et al., 2022).

Where *T*_*H*_ is hot end temperature, *T*_*C*_ is cold end temperature, and *ΔT* is temperature difference, respectively. *ZT* is the average value of thermoelectric properties, which can be used as a dimensionless parameter to measure the properties of TE materials:

$$ZT=\left(S^{2}\sigma /\kappa_{e}+\kappa_{L}\right)T$$ (2)

where *S* is the Seebeck coefficient, *σ* is the conductivity of the material, *к*_*e*_ is electronic thermal conductivity, *к*_*L*_ is lattice thermal conductivity, and *T* is the absolute temperature. Obviously, the larger the *ZT* value, the better the thermoelectric properties of the material. However, there is an intricate relationship between the Seebeck coefficient, electrical conductivity, and thermal conductivity, making it difficult for the thermoelectric figure of merit to be significantly improved for a long time (Bell, 2008; Snyder and Toberer, 2011). Therefore, our primary goal is to improve the electrical transmission performance (*S* ^*2*^ *σ*) of the material and reduce its heat transmission performance (*к*) through the coupling regulation of electrical transmission and heat transmission. According to Wiedemann-Franz law *к*_*e=*_ *LσT*, where *L* is the Lorentz constant. It can be seen that there is a positive correlation between electronic thermal conductivity and electrical conductivity. Therefore, as long as the thermal conductivity of the lattice with a small correlation is reduced, the thermoelectric figure of merit can be effectively optimized (Biswas et al., 2012; Zhao et al., 2014a; Kim et al., 2015; Li et al., 2017). Figure 2 summarizes the *ZT* statistical results of some typical TE materials in recent years (Ioffe et al., 1959; Cutler et al., 1964; Hicks and Dresselhaus, 1993; Horbach et al., 2001; Yang et al., 2001; Dashevsky et al., 2002; Kuznetsov et al., 2002; Hsu et al., 2004; Shutoh and Sakurada, 2005; Heremans et al., 2008; Poudel et al., 2008; Wang et al., 2008; Yang et al., 2008; Zhao et al., 2008; Xie et al., 2009; Du et al., 2011; Levin et al., 2011; Pei et al., 2011; Li et al., 2012; Liu et al., 2012; Chen et al., 2013; Gelbstein et al., 2013; Sui et al., 2013; Yamini et al., 2013; Yan et al., 2013; Hu et al., 2014; Lee et al., 2014; Tan et al., 2014; Wu et al., 2014; Zhao et al., 2014a; Zhao et al., 2014b; Zhong et al., 2014; Kim et al., 2015; Kraemer et al., 2015; Tan et al., 2015a; Tang et al., 2015; Huang et al., 2016; Liu et al., 2016; Tan et al., 2016a; Liu et al., 2017; Roychowdhury et al., 2017; Zhang et al., 2017; Li et al., 2018; Ren et al., 2018; Zhou et al., 2018; Xiang et al., 2019; Chong et al., 2020; Feng et al., 2020; Qin et al., 2020; Yvenou et al., 2020; Selimefendigil et al., 2021; Su et al., 2022).

Figure 2 shows that the thermoelectric materials studied mainly include tellurides, layered chalcogenide compounds, oxides, clathrates, Half-Heusler, skutterudite, and copper chalcogenides. For example, telluride itself has good low thermal conductivity and is potential thermoelectric material. Kim et al. (2015) synthesized Bi_0.5_Sb_1.5_Te_3_ by liquid-phase compression. The dense array of dislocations formed at the low-energy grain boundaries effectively scattered intermediate-frequency phonons, leading to a significant decrease in lattice thermal conductivity. The full-spectrum phonon scattering significantly increases the ZT to 1.86 at 320 K. Alkaline Earth oxides, such as cobalt compounds, have weak crystal symmetry and are also characterised by layered materials, which leads to a decrease in the lattice thermal conductivity of the material (Tang et al., 2015). The Cu_2_Se of the copper chalcogenide has a layered monoclinic crystal structure at low temperatures, and its symmetry is also poor. At the same time, Cu ions can migrate freely in the sublattice like a liquid at high temperatures. The liquid-phase metal ions induce a specific deformation of the crystal structure and a significant increase in asymmetry, which facilitates the acquisition of solid anharmonicity and increases the phonon scattering ability, while having no effect on the electron mobility (Gelbstein et al., 2013). Zhong et al. (2014) used DC hot pressing process to prepare layered flake Cu_1.94_Al_0.02_Se, found that under the linkage of the disordered movement of the liquid phase of the Cu ion layer and the low symmetry, a maximum *ZT* value of 2.62 appeared. And SnSe, BiCuSeO, GeTe, and other materials have also found great thermoelectric figures of merit in recent studies (Gelbstein et al., 2013; Sui et al., 2013; Zhao et al., 2014a; Li et al., 2018; Qin et al., 2020). These materials are expected to further improve thermoelectric performance through methods, such as further parameter optimization, nanostructure design, and tape structure engineering.

For today’s rapid development of modern technology, it is necessary to improve the *ZT* value of thermoelectric materials through different strategies. It has been shown that the strategy to improve the *ZT* value are mainly focused on the design of defect engineering. The first one is the energy band engineering design by adding atomic or second phase doping (Bell, 2008; Pei et al., 2011), which is done to optimize the carrier concentration and to increase the carrier mobility and thus obtain a more significant power factor. The doping process inevitably introduces lattice defects and distortions, which greatly affect the physical properties of the material. It is worth noting that the electric field, magnetic field, and light radiation can also stimulate and control the carrier concentration by cooperating with thermal energy transmission (Horbach et al., 2001; Du et al., 2011; Levin et al., 2011; Chen et al., 2013; Kraemer et al., 2015). The second is to rely on phonon engineering through nanostructures of different scales or mesoscale materials to reduce the lattice thermal conductivity *к*_*L*_ through phonon scattering at the interface and crystal plane (Heremans et al., 2008; Biswas et al., 2012; Tan et al., 2014). Here we review recent advances in several aspects of thermoelectric materials, such as carrier concentration, carrier mobility, effective mass, lattice thermal conductivity and evidence of low thermal conductivity materials, and then summarise the effects of electro-acoustic decoupling effects on thermoelectric materials, finding commonalities in these materials through the above sections and refining rational design strategies for exploring materials with high thermoelectric efficiency.

## Adjustments of Carrier Parameters to Improve *ZT*

### Carrier Concentration

The fundamental challenge of high *ZT* thermoelectric material design stems from *S*, *σ*, and *к*. Through the strong correlation of carrier concentration n, the energy level can generally be adjusted by controlling the doping. Assuming that the dopant does not significantly change the scattering or band structure, the Seebeck coefficient can be derived from (Cutler et al., 1964):

$$S=\frac{8\pi^{2}k_{B}^{2}}{3eh^{2}}m^{*}T{\left(\frac{\pi }{3n}\right)}^{2/3}$$ (3)

Among them, *k*_*B*_ is Boltzmann’s constant, m* is the effective carrier mass, *h* is Planck’s constant, and *n* is the carrier concentration. In semiconductor thermoelectric materials, according to the Kane energy band theory, the Hall carrier concentration can be expressed as (Ioffe et al., 1959; Cutler et al., 1964):

$$n_{H}=\frac{1}{eR_{H}}=A^{-1}\frac{N_{v}{\left(2m_{b}^{*}k_{B}T\right)}^{3/2}}{3\pi^{2}h^{3}}F_{0}^{3/2}$$ (4)

where *N*_*v*_ is the degree of degeneration, ^*0*^ *F*_*0*_ is the Fermi integral function, which is the reduced Planck constant. Although the carrier concentration *n* has an inverse relationship with the Seebeck coefficient, the increase of *n* has a positive effect on the energy band degeneration and the increase of effective mass. Therefore, the current strategy of optimizing carrier concentration usually adopts the increasing carrier Concentration method to improve thermoelectric performance (Dashevsky et al., 2002; Kuznetsov et al., 2002; Pei et al., 2011).

**FIGURE 3:**
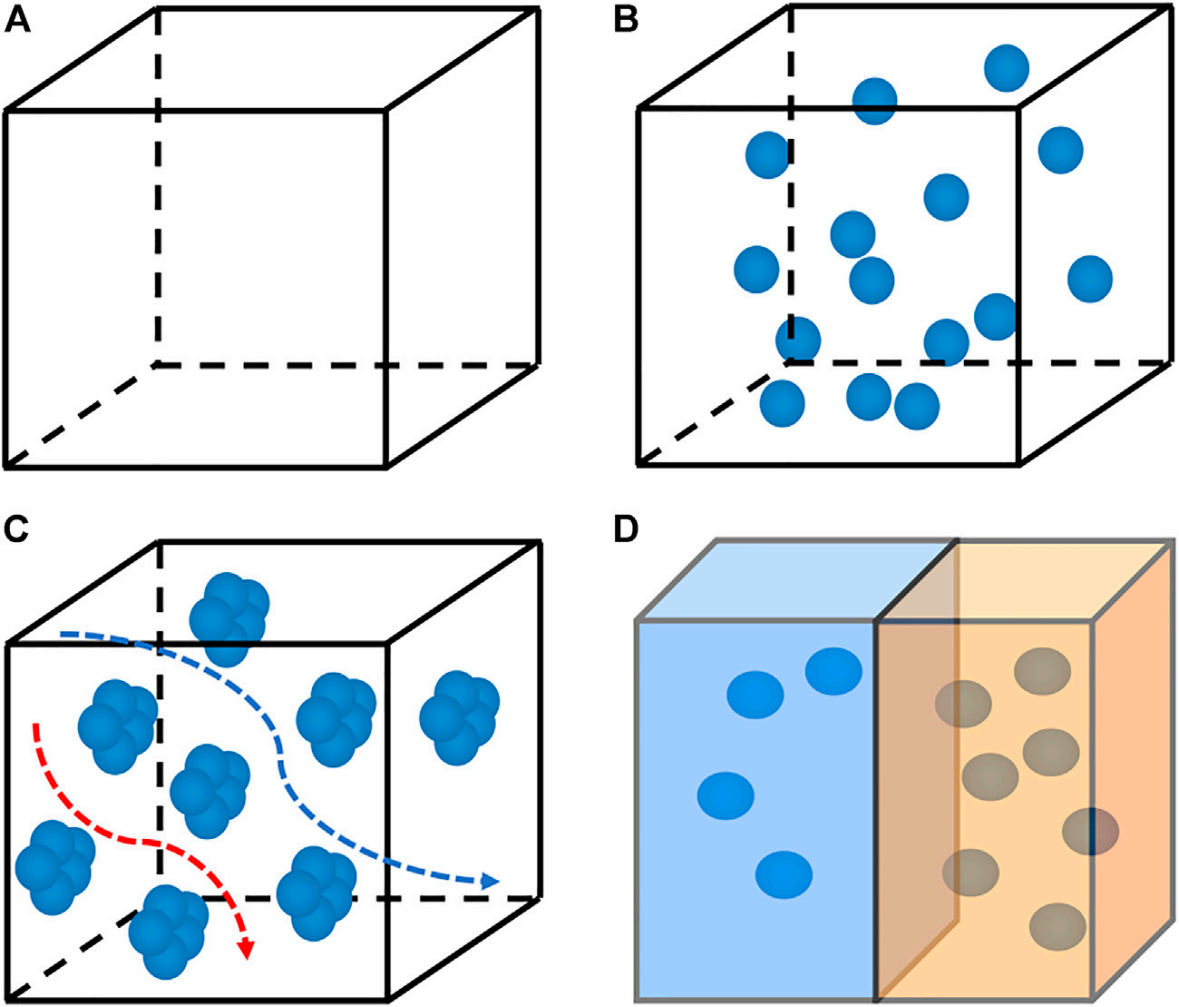
The schematic diagram of changing the carrier concentration by different doping methods; **(A)** undoped; **(B)** uniform doping; **(C)** modulated doping; **(D)** gradient doping.

**FIGURE 4:**
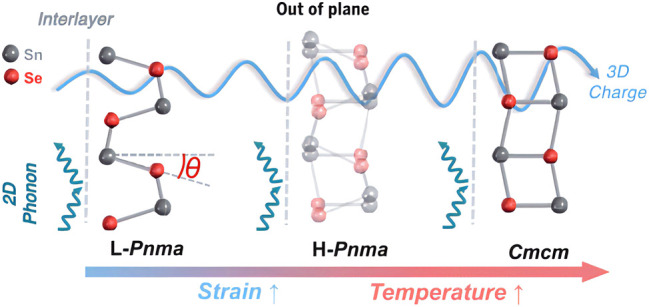
3D electron-2D phonon transport properties outside the n-type SnSe layer: Layered SnSe crystals use their layer structure to suppress (lattice vibration) phonon transport, but electrons different from phonons can tunnel through overlapping electron orbital transport (Su et al., 2022).

The main strategy for optimizing the carrier concentration is doping, such as modulation doping, uniform doping, and gradient doping (Figure 3). Feng et al. (2020) systematically revealed the theoretical origin of high average *ZT* in GeTe-based alloys uniformly doped with Pb through theoretical simulations and experimental tests on GeTe-based alloys. On the one hand, the intrinsic high hole carrier density is reduced to a rough optimal range, and then the carrier density is fine-tuned by Pb doping. The lead-doped energy band convergence can maintain a higher power factor. On the other hand, lead causes a decrease in carrier density and can significantly reduce *κ*, and the combination of the two is almost the same. PF results in a maximum *ZT* at the corresponding carrier density, so that the average *ZT* is about 1.5 in the 300–773 K range and the peak *ZT* is about 2.0 at 573 K, indicating that Pb doping can be used over a wide temperature range (e.g., 300–800 K), rather than only for TE applications at higher temperatures (e.g., T > 600 K) (Feng et al., 2020). In the case of uniform doping, the carrier concentration is generally independent of temperature, so the best thermoelectric performance cannot be guaranteed in the entire temperature range. One way to deal with this problem is to use gradient doping. By integrating two or more different segments, a common method of preparing this graded material is the so-called spark plasma sintering or hot-pressed powder layer. Each powder layer has different carrier concentration (Dashevsky et al., 2002; Selimefendigil et al., 2021). Zhao et al. (2014c) take advantage of the low thermal conductivity of layered materials in the out-layer direction. The migration of carriers between layers is improved in the out-layer direction by adjusting the symmetry of the crystal structure, thereby promoting electron tunneling in the inter-layer direction. At 600–800 K, SnSe has a continuous phase transition process from low-symmetry Pnma (L-Pnma) phase to high-symmetry Pnma (H-Pnma) phase, and then to high-temperature high-symmetry Cmcm phase. In addition to temperature, introducing stress into SnSe crystals by doping/solid solution can also adjust the symmetry of the crystal structure, thereby optimizing the thermoelectric properties of the material, as shown in Figure 4 (Su et al., 2022).

However, with prolonged use, the initial carrier concentration gradient in the gradient material may decrease or disappear due to the homogenization effect induced by diffusion, thereby reducing the conversion efficiency. To solve this limitation, using the temperature dependence of the solubility of certain specific dopants (Yamini et al., 2013), a carrier concentration n is controlled only by temperature, and its gradient can be created in a single material. A well-known example is Cu, Ag, and excess Pb. They have negligible solubility in PbTe at room temperature but have high solubility at high temperatures (Bergum et al., 2011). Lidong Zhao et al. synthesized an n-type SnSe crystal with a two-dimensional layered structure using the temperature gradient method and bromine doping method. The electron doping promotes the hybridization of delocalized electrons and realizes the tunneling of electrons between the n-type SnSe layers. This “two-dimensional phonon/three-dimensional charge” transfer characteristic greatly improves the thermoelectric properties of n-type SnSe crystal materials, with a *ZT* as high as 2.8 (Chang et al., 2018). This temperature-dependent doping is reversible in the heating-cooling process and eliminates the diffusion problem of gradient doping, making it more suitable for practical applications. Polyatomic doping takes advantage of the synergistic effect to have a more significant change in carrier concentration (Zhu et al., 2002). Lixia Zhang et al. fabricated the Yb-filled CoSb_3_ skutterudites with an ultra-high Yb filling fraction by melt-spinning methods, and they found a high ZT value of about 1.3 was achieved at 823 K for the nominal composition Yb_0.4_Co_4_Sb_12_. An ultra-low lattice thermal conductivity approaching the glass limit was achieved due to the enhanced rattling effect from the ultra-high Yb filling fraction and extra phonon scattering from the ordered superstructures, nano-scale inhomogeneous Yb fillers, and high-density lattice strain caused by the ordered and modulated fillers (Ren et al., 2021). Shi et al. (2011) synthesized CoSb_3_ with a variety of co-fillers such as Ba, La, and Yb. Finally, from single filling to double filling, and finally to multiple fillings to achieve continuous growth of *ZT* value, the maximum *ZT* at 850 K was increased to 1.7. Therefore, for thermoelectric material systems with different characteristics, it is particularly important to select a suitable doping method (the most current method is temperature-dependent doping) to optimize the carrier concentration in a broader temperature range.

### Carrier Mobility

**FIGURE 5:**
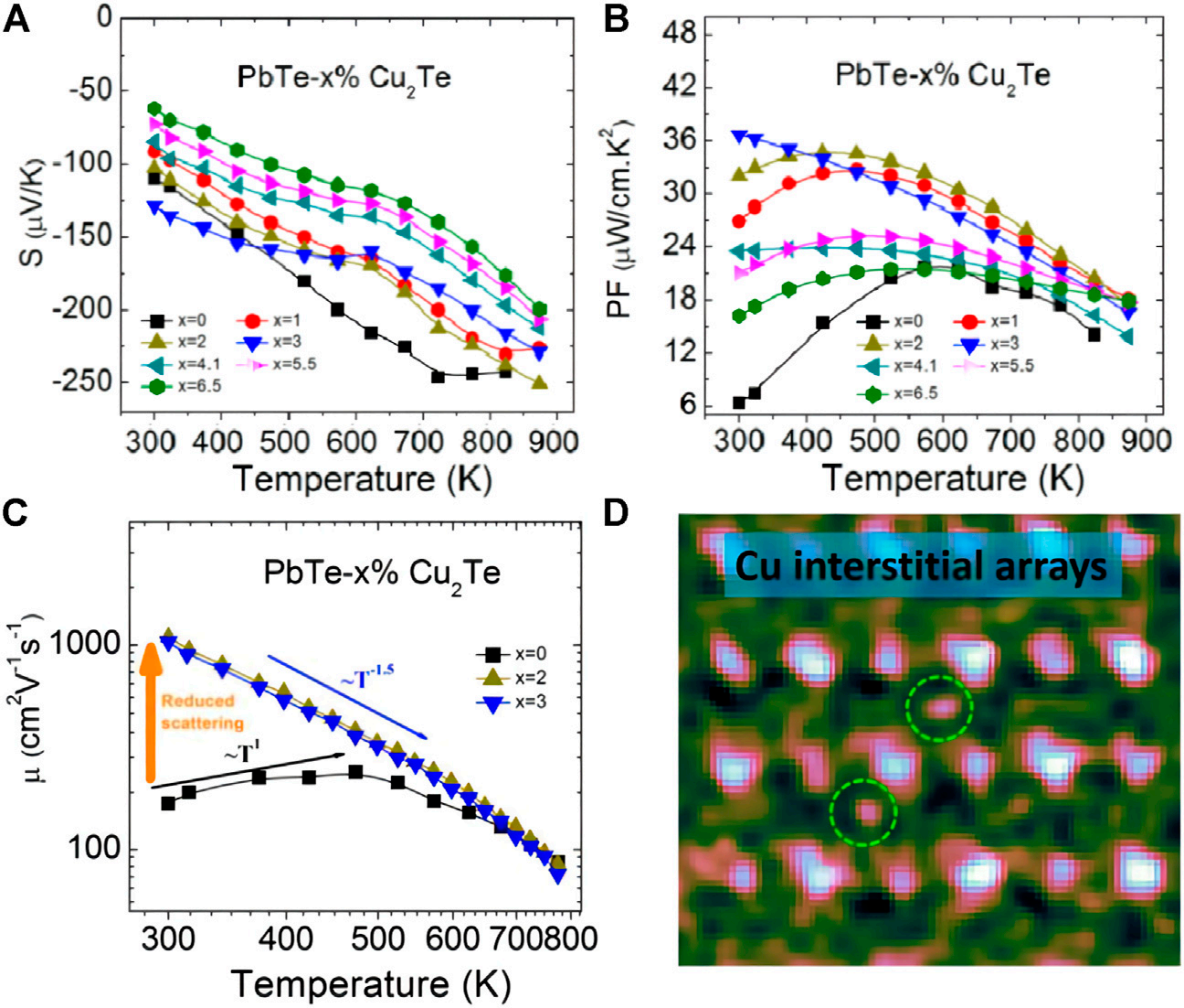
**(A)** Elimination of lead vacancies can reduce carrier scattering and significantly increase carrier mobility; **(B)** PbTe-Cu_2_Te electrical transport properties change with temperature; **(C)** PbTe-Cu_2_Te power factor change with temperature; **(D)** High magnification STEM HAADF image of Cu gap array (enlarged) (Xiao et al., 2017).

The improvement of carrier mobility is mainly through introducing impurity atoms in the intrinsic atomic vacancies. In most cases, the increase of m* will lead to a decrease in carrier mobility, and it will reduce the conductivity (Ioffe et al., 1959):

$$\mu =\frac{e\tau }{m^{*}}$$ (5)

where *τ* is the relaxation time, so the improvement of carrier mobility also has a positive effect on the increase of *ZT*. Generally speaking, the smaller the electronegativity difference between the elements of the compound, the greater the mobility of the material, such as PbTe, Bi_2_Se_3_, etc. Lidong Zhao et al. further studied the effect of copper doping on n-type PbTe. The effective filling of Cu reduces the scattering of carriers in the gaps of Pb crystals, resulting in considerable carrier mobility and a high power factor (Xiao et al., 2017). The PbTe-2% Cu_2_Te reaches a peak value of *ZT* ∼ 1.5 at 723 K, and the *ZT*_*ave*_ ∼ 1.0 of the n-type PbTe system achieves the highest value in history, as shown in Figure 5.

Studies have shown that to improve carrier mobility, three-dimensional modulation doping (mainly under low-temperature conditions) can effectively increase the *ZT* value of some important thermoelectric materials (Zebarjadi et al., 2011; Koirala et al., 2013; Pei et al., 2014). The modulated doped sample is a two-phase composite material composed of undoped and heavily doped. Undoped original samples have low carrier concentration but high carrier mobility, while uniformly doped and heavily doped samples have high carrier concentration but low carrier mobility. The Fermi level of undoped samples is usually in the middle of the energy gap, and the Fermi level of heavily doped samples goes deep into the conduction band (n-type doping) or valence band (p-type doping). With the energy imbalance between the undoped phase and the heavily doped phase, the carriers in the sample overflow from the heavily doped area to the undoped site, and this process enhances the carrier mobility (Valset et al., 2012).

In addition to modulating doping, the texture structure of the material can be used as another feasible way to improve carrier mobility, especially for some materials with anisotropic structures. In various heterostructures, the carrier mobility may only be higher in some specific crystal orientations (Zhao et al., 2014a). Li et al. (2018) proposed a method of high-efficiency thermoelectric materials that optimizes both electrical and heat transport performance. In (Bi, Sb)_2_Te_3_, the two-step sintering process with excess Te can increase mobility and reduce thermal conductivity at the same time. The two-step sintering process reduces the grain boundaries and defects that seriously hinder the transport of charge carriers, thereby increasing the mobility, and the maximum *ZT* of Bi_0.4_Sb_1.6_Te_3_ can reach 1.3. Studies have shown that the two-step sintering technology is an effective method, which can control the existence and distribution of element inhomogeneity, so that the carrier transport and phonon transport have a special directionality. This has a specific guiding significance for other thermoelectric material systems with similar characteristics (Cha et al., 2019; Pan et al., 2019).

## Improvement of Effective Quality

The effect of the local increase of the density of states on the Seebeck coefficient is given by Mott’s expression (Heremans et al., 2008). When semiconductors are doped or heavily doped with metals, according to the Mahan-Sofo theory, at a given carrier concentration, a high total density of states effective mass (*m*_*d*_ *) will determine a high Seebeck coefficient (Pei et al., 2012a), and then increase the *ZT* value of the material. To increase local DOS in a narrow energy range, md* can be effectively increased. Since the *ZT* value is related to the conductivity, the carrier concentration n cannot be too low. Most high-performance thermoelectric materials are heavily doped semiconductors, and the optimal carrier concentration *n*_*opt*_ varies with (m*T)^3/2^. Therefore, *n*_*opt*_ can also be achieved by tuning the effective quality *m*_*d*_ * (Cha et al., 2019). According to *m*_*d*_ * = *N*_*v*_^*2/3*^ *m*_*b*_ *, degeneracy (*N*_*v*_) and effective mass (*m*_*b*_ *) play a decisive role in *m*_*d*_ *. In most cases, the increase in *m*_*d*_ * is due to the increase in *m*_*b*_ * (Pei et al., 2012b; Dutta et al., 2020), with the increase of the effective mass *m*_*b*_ * will result in a decrease in the carrier mobility *μ* and a decrease in the material conductivity (Tan et al., 2016b). Energy band engineering causes the density of states distortion by doping or changing symmetry, using energy band degeneration, increasing resonance states, hybridizing the energy gap, and doping Kondo atoms to change its effective mass essentially (Suwardi et al., 2019).

**FIGURE 6:**
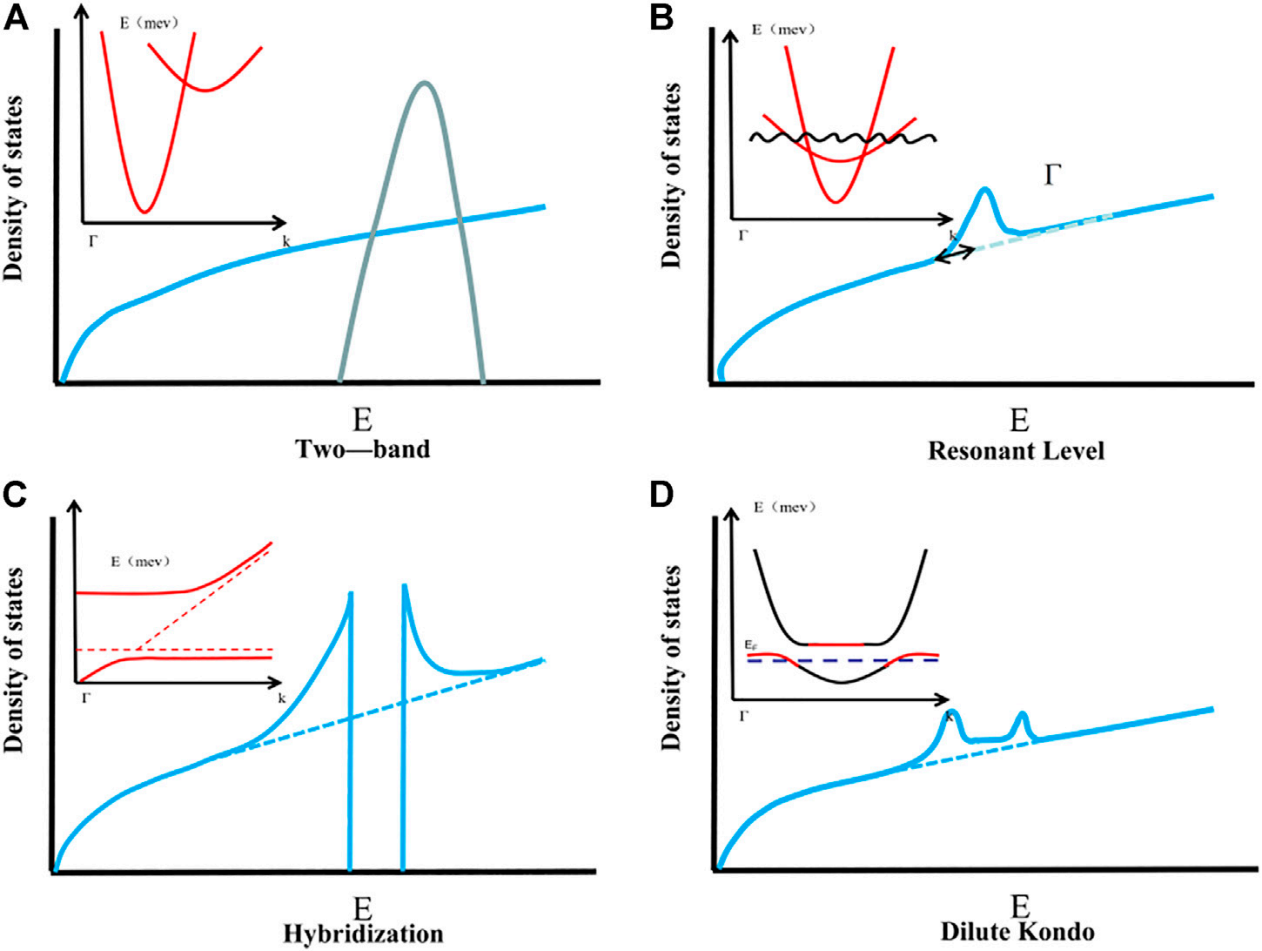
Four ways to improve effective mass: **(A)** Degenerate energy band; **(B)** Increase resonance state; **(C)** Hybrid energy gap; **(D)** Doping Kondo atoms.

High energy band degeneracy is of great significance in thermoelectric materials because it can produce higher *m*_*d*_ * (as shown in Figure 6A), resulting in a more significant Seebeck coefficient, and has no deleterious effect on carrier mobility. *N*_*v*_ is closely related to the symmetry of the crystal structure. Low symmetry has very low *к*_*L*_ and poor thermoelectric performance. The current superior TE materials usually have a high symmetry crystal structure and symmetry-related multi-valley carrier structure (Zhang et al., 2014). To obtain more equivalent degenerate valleys and peaks, the *N*_*v*_ and Seebeck coefficients can be increased. For example, Zhao’s team found that due to the layered change of the crystal structure in the SnSe crystal structure, different *N*_*v*_ causes different *m*_*b*_ * in different directions, and the *ZT* value changes (Zhao et al., 2016). Then, Jiawei Zhang designs a constrained cubic structure by coexisting a long-range cubic frame and a partial short-range cubic lattice distortion (Zhang et al., 2014). By controlling the crystal structure of diamond distortion (Li et al., 2018), achieves low symmetry with convergence, while reducing the lattice thermal conductivity and increasing *ZT* values, it is shown that the symmetry breaking with degeneracy is small, and the high simplification and low lattice thermal conductivity can be realized simultaneously. This method of reconstructing the approximate cubic structure to obtain a highly symmetrical crystal structure is considered to be a feasible approach to obtaining a larger *N*_*v*_ and a higher Seebeck coefficient.

**FIGURE 7:**
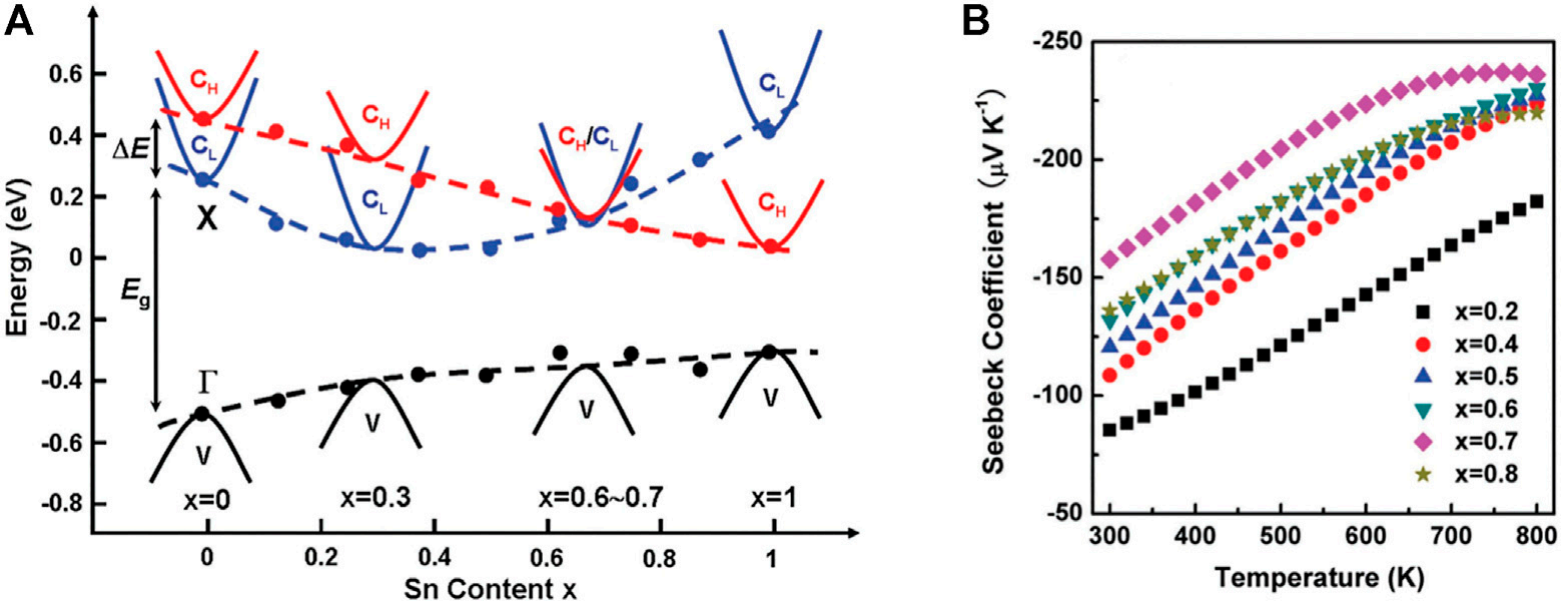
**(A)** The relationship between the tin content in the Mg_2_Si_1−x_Sn_x_ solid solution and the relative position of the weight and light transmission bands and the highest valence band; **(B)** the temperature dependence of the Seebeck coefficient of the n-type Mg_2_Si_1−x_Sn_x_ solid solution (Su et al., 2017).

Another effective method to improve *N*_*v*_ is to converge the different bands in the Brillouin region within the *nk*_*B*_ *T* of each other’s energy. The valley number in the conduction band and the peak number in the valence band increase, so that the mobility is not affected by *N*_*v*_. For example, Su et al. (2017) regulates *N*_*V*_ according to the formation of Mg_2_Si_1−x_Sn_x_ in the solid solution, when the concentration of Sn is close to *x* = 0.7, the band edges of the two conduction bands are guaranteed to overlap, and the band convergence is realized, as shown in Figure 7A. Due to the increase of *m*_*d*_ *, the Seebeck coefficient reaches its maximum value (as shown in Figure 7B), resulting in abnormally high TE performance at 700 K (*ZT* = 1.3). It is proved that by doping, the band structure with multiple extreme values can be formed at the top or bottom of the valence band, or the convergence of light band and heavy band can increase the degeneration of the band, so as to obtain better thermoelectric performance.

The introduction of the resonance state can cause the density of states to be distorted (as shown in Figure 6B). If the Fermi energy *E*_*F*_ falls near the center of the resonance band, the PbTe density of states is distorted in Figure 6B, resulting in a large increase in the Seebeck coefficient. It is mainly achieved by doping, usually because after the resonant impurity atoms are introduced into the complete periodic structure, the same energy level and different energy states are superimposed so that the energy band of the doped impurity falls below the edge of the valence band or the host above the edge. In general, the lower the resonance level, the greater the distortion of the electronic density of states, and the Seebeck coefficient will increase (Dutta et al., 2020). Heremans et al. (2008) used PbTe doping with Ti and found that the electron DOS was enhanced near the Fermi level. Later, Tan et al. (2016a) used Na atoms to replace vacancies and defects in PbTe, which once again proved that doping can actually increase the effective mass of the density of states and increase the *ZT* value. However, Pei et al. (2012b) improved the effective mass by doping La in PbTe. They found that the effective mass is temperature-dependent, and the increase in the effective mass leads to an increase in the Seebeck coefficient, but it actually increases the *ZT* value significantly in the lower temperature range (Pei et al., 2012b), slightly deviating from the previous rule. Therefore, the core law of changing the effective mass of the density of states through the introduction of the resonance state to increase the *ZT* value needs further exploration.

The hybrid energy gap is another way to increase the effective mass of the density of states, as shown in Figure 6C. However, this method has great limitations on the change of the density of states. It will cause the carrier mobility of the material to drop to zero in a certain energy interval, and make the effective mass return to zero, which is fatal to the thermoelectric performance. However, the hybrid energy gap in its vicinity can effectively increase the effective mass of its density of states, and enhance the thermoelectric performance. For example, Tan et al. (2019) introduced an internal axis nanostructure into n-type PbTe, and they performed CdTe alloying and Sb doping on it, which enhanced the effective mass of state density by expanding the band gap, and increased the Seebeck coefficient. Improving the thermoelectric performance is also an effective method to enhance the Seebeck coefficient.

The Kondo effect may be triggered in some thermoelectric systems doped with magnetic atoms. The study found that the electrical conductivity of the material is higher when the Kondo temperature is near. The coupling of these Kondo atoms with nearby electrons will form a larger-scale Kondo cloud, which has an enhancement effect on the density of states, as shown in Figure 6D. Lu et al. (2015) maintains the additional valence band of the conductive spin-split Ni state by adding Ni-doped tetrahedral Cu_12_Sb_4_S_13_, electrons fill holes in the valence band, and the resulting Kondo effect increases the thermoelectric performance by more than 30%. Qiu et al. (2014) used Fe doped Cu_2_S at 900 K, the maximum *ZT* reaches 1.2, because Fe atoms are randomly distributed on the substrate to form a Kondo cloud, which leads to higher stability than Cu_2_S (Tan et al., 2019). However, the Kondo effect will also reduce the electrical conductivity of the material when the Kondo temperature is far away, or the Kondo cloud-scale is too large, which is not suitable for increasing the *ZT* value. Therefore, it is generally used to dope Kondo atoms, while ensuring that the system application range is at the Kondo temperature. In the vicinity, the *ZT* value is guaranteed to increase. However, the current interpretation of the Kondo effect is still in its infancy, and there is a wide range of design prospects in the later period.

There have been extensive studies on improving the Seebeck coefficient by adjusting the effective mass through energy band engineering, but the improvement of the effective mass also means the reduction of carrier mobility, so blindly improving the effective mass is not necessarily beneficial to increasing the *ZT* value, even Some scholars believe that reducing the effective mass of energy bands in some systems can increase the *ZT* value (Pei et al., 2012a). Therefore, how to balance the relationship between effective mass and carrier mobility to achieve the maximum *ZT* value still needs further investigation and verification.

## Reduction of Lattice Thermal Conductivity

The lattice thermal conductivity *к*_*L*_ is an independent influencing factor, and it is the most direct and effective means to optimize the thermal power figure.

According to the lattice thermal conductivity formula:

$$\kappa_{L}=\frac{1}{3}C_{v}V_{g}l=\frac{1}{3}C_{v}V_{g}^{2}\tau$$ (6)

(where *C*_*v*_ is the constant volume heat capacity, *V*_*g*_ is the group velocity of the phonon vibration mode, *l* is the mean free path, and *τ* is the phonon relaxation time) affects the lattice thermal conductivity. The main factors of the rate are heat capacity, speed of sound, and relaxation time. Therefore, by reducing any parameter, the lattice thermal conductivity can be reduced. The atomic point defects formed by element substitution and the nanostructures formed by the nucleation and growth of the second phase can significantly scatter short-wave phonons and medium-wave phonons, respectively. It is precise because the new structure generates an increased probability of phonon collisions, reduces the mean free path of phonons and the relaxation time of phonons, and enhances phonon scattering. And when the size of the defect in the material is close to the mean free path of the phonon, it will have a greater scattering effect on the phonon (Zhao et al., 2014c). According to the thermal conductivity model proposed by Klemens and Callaway (Callaway, 1959; Klemens, 1960), the degree of scattering reduction can be written by the scattering parameter evaluation (*Γ*) as:

$$\Gamma =x\left(1-x\right)\left[{\left(\frac{\Delta M}{M}\right)}^{2}+\varepsilon {\left(\frac{a_{disorder}-a_{pure}}{a_{pure}}\right)}^{2}\right]$$ (7)

where *x* is the doping fraction, *ΔM/M* is the rate of change of relative atomic mass, *α*_*disorder*_ and *α*_*pure*_ represent the lattice constants of disordered and pure alloys, and *ε* is an elasticity-related adjustment parameter. Here we can find that maximizing *Γ* will result in the lowest lattice thermal conductivity. Generally, higher doping fractions, heavy element doping, and greater lattice mismatch can be used to reduce irritation. Next, we use several scattering mechanisms such as point defect scattering, dislocation scattering, interface scattering, and phonon resonance scattering to explain the influence of the above factors.

### Point Defect Scattering

**FIGURE 8:**
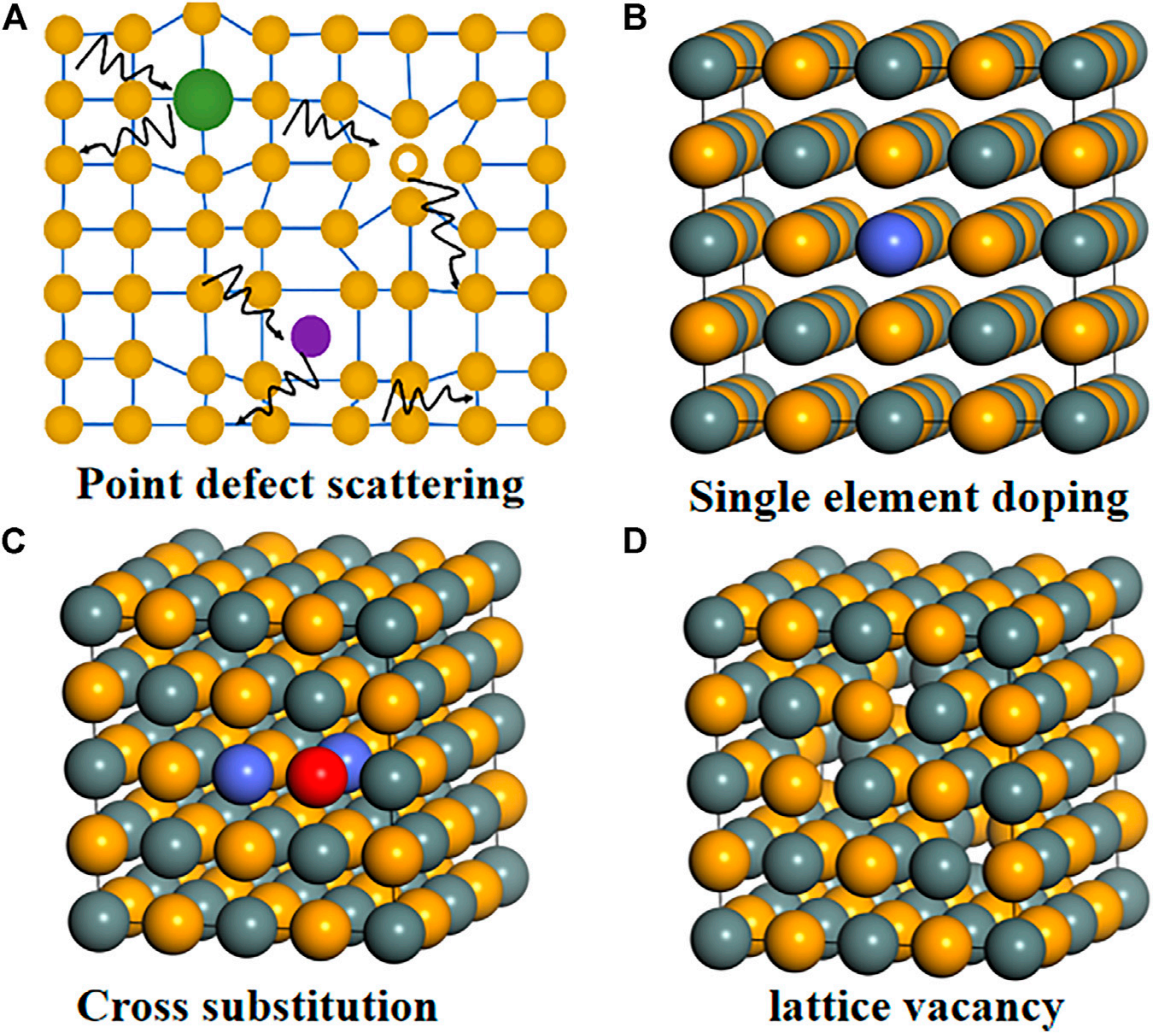
Schematic diagram of point defect scattering and atomic-scale doping methods; **(A)** Schematic diagram of point defect scattering; **(B)** single-atom doping; **(C)** polyatomic doping; **(D)** lattice vacancy.

Introducing lattice point defects into the matrix lattice is an effective way to reduce the thermal conductivity of the lattice, as shown in Figure 8A. This is because phonons are more likely to be scattered by point defects rather than electrons, and thus have less impact on carrier mobility. Figures 8B–D show typical doping methods commonly used in thermoelectric materials research, namely single element doping, cross-substitution, and formation of lattice vacancies. In the case of single doping, the dopant can be the same valence as the main element, resulting in lattice disorder, or it can be heterovalent, so as to control the carrier concentration. However, this doping causes the system charge imbalance, which may hinder the reduction of *к*_*L*_ if done alone.

To improve the solubility of different elements, cross-substitution is generally used. Cross-substitution refers to the pair-to-pair substitution of one or more main elements from other groups in the periodic table, while keeping the total number of valence electrons unchanged (Tan et al., 2013; Tan et al., 2015b).

Yu et al. (2018a) designed thermoelectric materials by isoelectronic substitution of the atom Ta for the atom Nb. By using isoelectronic substitution of atoms with similar size and chemical properties but the large mass difference, the lattice thermal conductivity of the alloy was greatly suppressed, but good electrical properties were maintained. As a result, the *ZT* reaches a peak of 1.6 at a Ta content of 0.4. It shows that the point defects caused by the substitute atoms will change the thermal conductivity of the lattice (Yu et al., 2018a). It is worth noting that the cross-substitution not only greatly affects the transport of phonons, but may also change the electronic structure (mainly the band gap), providing other ways to adjust the thermoelectric properties of the matrix material. Because only when these two heterogeneous elements exist together in a specific ratio, the amount of them entering the matrix will be greater. We call this effect synergistic alloying. In the Pb_0.98_Na_0.02_Te-8% SrTe sample, the *ZT* value is 2.5 at 923 K, and the average *ZT* value is 1.67 in the range of 300–900 K. It is further proved that by optimizing the Na concentration, a *ZT* of 2.5 can be obtained at a lower temperature of about 800 K (Xu et al., 2020a). Alloying is currently the most widely used method to enhance the scattering of point defects, in Bi_2_Te_3_ (Hu et al., 2014; Chen et al., 2015), Pb(Te, Se) (Pei et al., 2011), GeTe (Chen et al., 2020) and Half-Heusler (HH) alloys (Xue et al., 2016; Shen et al., 2018; Yu et al., 2018b) have applications.

In addition to alloying, vacancies and interstitial atoms also belong to a relatively special point defect scattering mechanism (Xue et al., 2016). Jiaqing He introduced double-vacancy defects by doping BiI_3_ (Xu et al., 2020b) to improve the thermoelectric performance of Sb_2_Te_3_(GeTe)_17_. The maximum value of *ZT* is 2.2 at 723 K. There are nearly 20% intrinsic Nb vacancies in the HH alloy Nb_0.8_CoSb, which makes the Nb_0.8_CoSb alloy have a relatively low lattice thermal conductivity (Xia et al., 2018). The same phenomenon exists in thermoelectric materials such as Cu_2_SnSe_4_ (Li et al., 2016). Like vacancies, interstitial atoms can also be generated by a solid solution method, but only compounds with a larger cation-anion ratio are applicable. The cations of such compounds need to have a smaller radius to fit the interstitial sites. Taking the solid solution (SnTe)_1−x_ (Cu_2_Te)_x_ as an example (Li et al., 2017; Zheng et al., 2017), its lattice thermal conductivity is as low as 0.5 W m^−1^ K^−1^, which is close to the minimum lattice thermal conductivity limit of SnTe (About 0.4 W m^−1^ K^−1^).

### Dislocation Scattering

**FIGURE 9:**
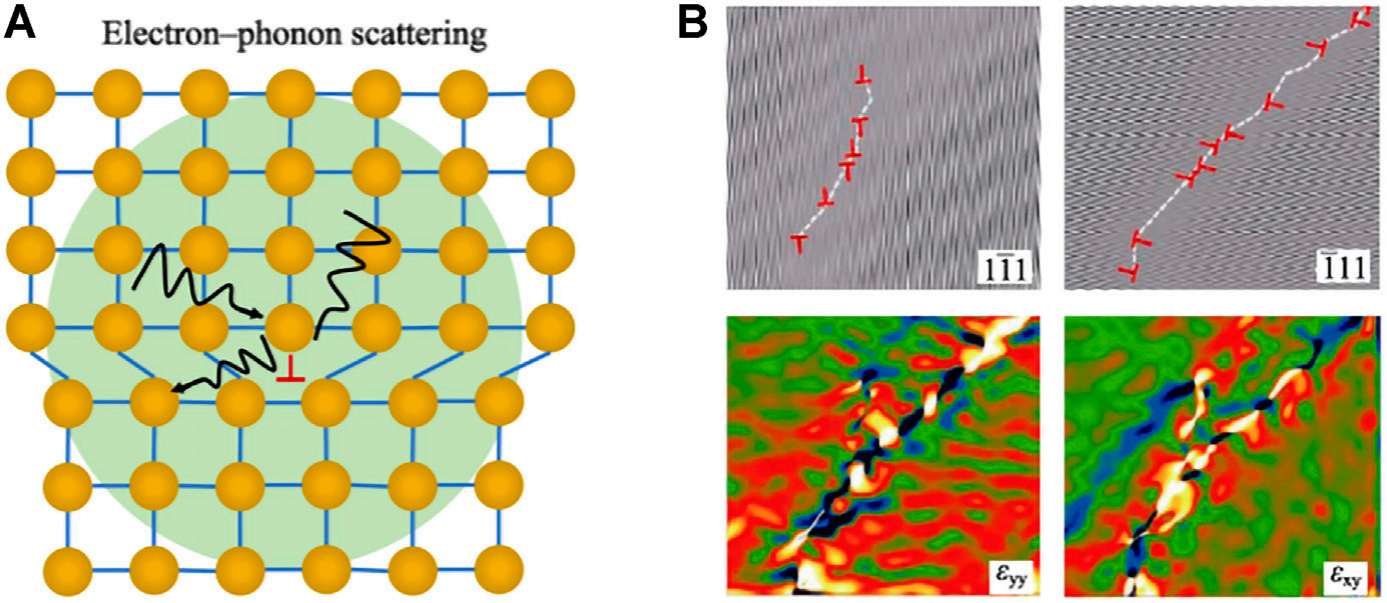
**(A)** Schematic diagram of dislocation scattering; **(B)** IFFT diagram of dislocation in Mg_2_Si_0.5_Sb_0.5_ and the corresponding stress scan diagram (Xin et al., 2017).

Dislocations are a typical line defect, especially in metal materials. Dislocations and strain fields will scatter mid-and high-frequency phonons, so dislocations can effectively reduce the free path of phonons (Figure 9A). Kim et al. (2015) reported that by forming a high-density dislocation array at the grain boundary by extrusion, the lattice thermal conductivity of the Bi_0.5_Sb_1.5_Te_3_ material could be significantly reduced, which is a dense dislocation array pair. The result of phonon scattering. In addition, dislocations can also be introduced through the precipitation of the second phase, where dislocations can play a role in reconciling the lattice mismatch at the phase boundary (Hu et al., 2015).

In solid solution materials, due to the accumulation of vacancies, adjacent spaces collapse under external conditions to form vacancy dislocations. At the same time, vacancies will also spontaneously reduce energy. The released energy can promote dislocations to climb. The climbing motion of dislocations will increase the density of dislocations, and finally produce high-density and uniformly distributed dislocations in the crystal grain, thereby reducing *к*_*L*_. In Mg_2_Si_1−x_Sb_x_ materials (Xin et al., 2017), high-dose alloying of Sb has a large number of Mg vacancies, making the vacancy concentration much higher than the equilibrium vacancy density, and the excess Mg vacancies spontaneously aggregate to form dislocations, as shown in Figure 9B. In Mg_2_Si_0.5_Sb_0.5_, the dislocation density is as high as 2.8 × 10^16^ m^−2^. Besides, similar phenomena were also observed in the Na_y_Eu_0.03_Pb_0.97−y_Te system (Chen et al., 2017). Studies have shown that with the increase of Na doping, the main microscopic defects in the system gradually change from point defects to dislocations. Dislocation scattering reduces the lattice thermal conductivity of PbTe to below 0.4 W m^−1^ K^−1^.

### Interface Scattering

**FIGURE 10:**
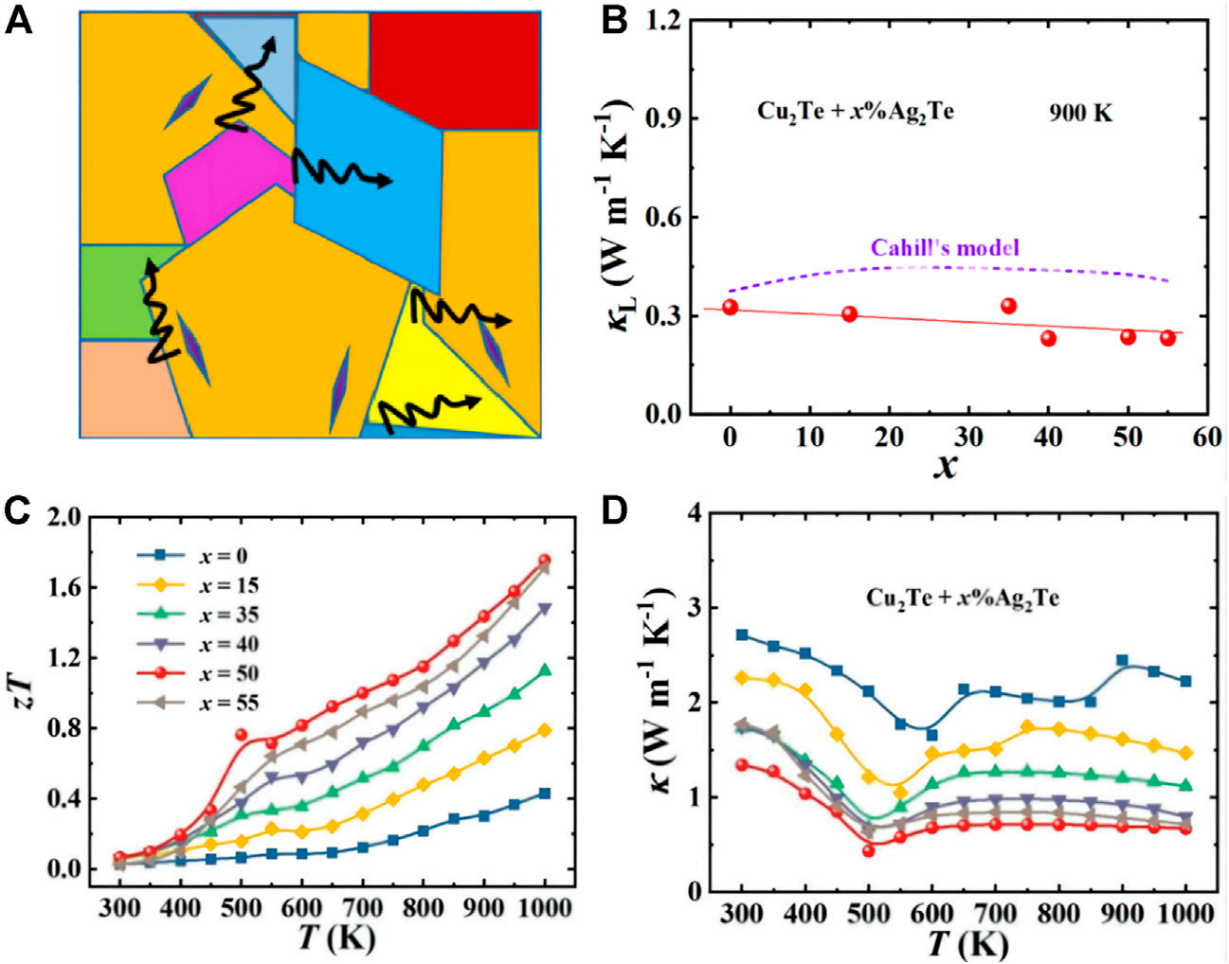
**(A)** Schematic diagram of interface scattering; **(B)** lattice thermal conductivity *κ*_*L*_ at 900 K for all samples of Cu_2_Te + *x*% Ag_2_Te; **(C)** and **(D)** are Cu_2_Te + *x*% Ag_2_Te(*x* = 0, 15, 35, 40, 50, and 55) thermal conductivity (*к*), thermoelectric figure of merit (*ZT*) varies with temperature (Zhao et al., 2019).

Because atomic defects and dislocation defects can effectively scatter low- and mid-wavelength phonons, there are still some long-wavelength phonons that are not scattered and affect the thermal conductivity of the lattice. In polycrystalline materials, grain boundaries or phase boundaries can effectively scatter low-frequency phonons, and the scattering rate is inversely proportional to the grain size. Therefore, the effective method to effectively scatter long-wavelength phonons is to introduce micro-nano structures (Girard et al., 2010). The mechanism of action is shown in Figure 10A. In particular, nanostructures have been proven to be an effective method to enhance *ZT* by placing suitable nano-scale precipitates in the matrix to reduce *к*_*L*_, such as AgPb_m_SbTe_m+2_ (Hsu et al., 2004), NaPb_x_SbTe_2−x_ (Poudeu et al., 2006) and PbTe-Ag_2_Te (Zhao et al., 2013a). In addition, p-type PbTe_1−x_Se_x_ (Pei et al., 2011) and Tl-PbTe (Heremans et al., 2008) also have excellent thermoelectric properties, which come from the introduction of multiple valence bands and the density of states distortions in the valence band, respectively. Biswas et al. (2012), Liu et al. (2016), and others considered phonon scattering sources on all scales from the perspective of a layered approach, ranging from atomic-scale lattice disorder and nano-scale internal deposits to mesoscale crystals. The thermal conductivity of the crystal lattice is greatly reduced. Finally, the *ZT* of PbTe-SrTe is 2.2, and the *ZT* of Bi_0.88_Ca_0.06_Pb_0.06_CuSeO is 1.5, which proves that the grain boundary scattering is important in reducing the thermal conductivity. Zhao et al. (2019) introduced the Ag_2_Te phase into Cu_2_Te. Due to multiple phase transitions, they show complex temperature dependence in the temperature range of 300–1000 K. As the Ag_2_Te content increases, the interface scattering increases, and *к*_*L*_ decreases significantly (as shown in Figures 10B–D). At the same time, its highest *ZT* value reaches 1.8. It shows ultra-low lattice thermal conductivity at 900 K, and its value is about 0.3 W m^−1^ K^−1^ (Zhao et al., 2019).

Commonly used methods to enhance grain boundary scattering include ball milling (Li et al., 2015) and plasma sintering (Xie et al., 2018), etc., to obtain finer or smaller grains from coarser-grained crystal powders to increase the grain boundary area. Li et al. (2015) prepared nanostructured Na_0.02_Pb_0.98_Te samples with a grain size of 20–100 nm through high-energy ball milling and semi-solid powder processing. Through observation, they found that the interface between the particles can be clearly seen. The size (0.1–1 μm) of the pores, so there is stronger phonon scattering at the grain boundary, which reduces the lattice thermal conductivity *к*_*L*_ to 0.74 W m^−1^ K^−1^ at 623 K. Similarly, Yu et al. (2017) combined melt spinning, ball milling and spark plasma sintering processes to construct a microstructure containing a large number of 60° twin grain boundaries in the Bi_0.5_Sb_1.5_Te_3_ liquid alloy. The bilateral grain boundaries not only increase the current load mobility, and its scattering effect on phonons greatly reduces the thermal conductivity, so that *ZT* = 1.42 at 348 K (Yu et al., 2017).

In addition to the above methods, the process of separating the micro-nano phases to form twin crystals and dislocation network structures can also increase the grain boundary area, thereby enhancing phonon scattering and reducing thermal conductivity (Kim et al., 2015). Gelbstein et al. (2013) separated the p-type Ge_0.87_Pb_0.13_Te into two phases of GeTe and PbTe through spark plasma sintering, thereby generating the characteristics of multiple interfaces, enhancing the phonon scattering at the crystal plane, and achieving a relatively highly stable TE Performance.

It can be seen that defects of different sizes prepared by doping in materials and related physical and chemical methods can effectively scatter phonons of different wavelengths and different free paths, thereby reducing the thermal conductivity of the lattice. Therefore, the changes in energy band and structure (mass ratio and degree of distortion) caused by the coordinated effect of point defect scattering, dislocation scattering, and interface scattering will enhance the scattering of phonons at different wavelength levels simultaneously (Li et al., 2018; Lee et al., 2019).

### Resonance Scattering

**FIGURE 11:**
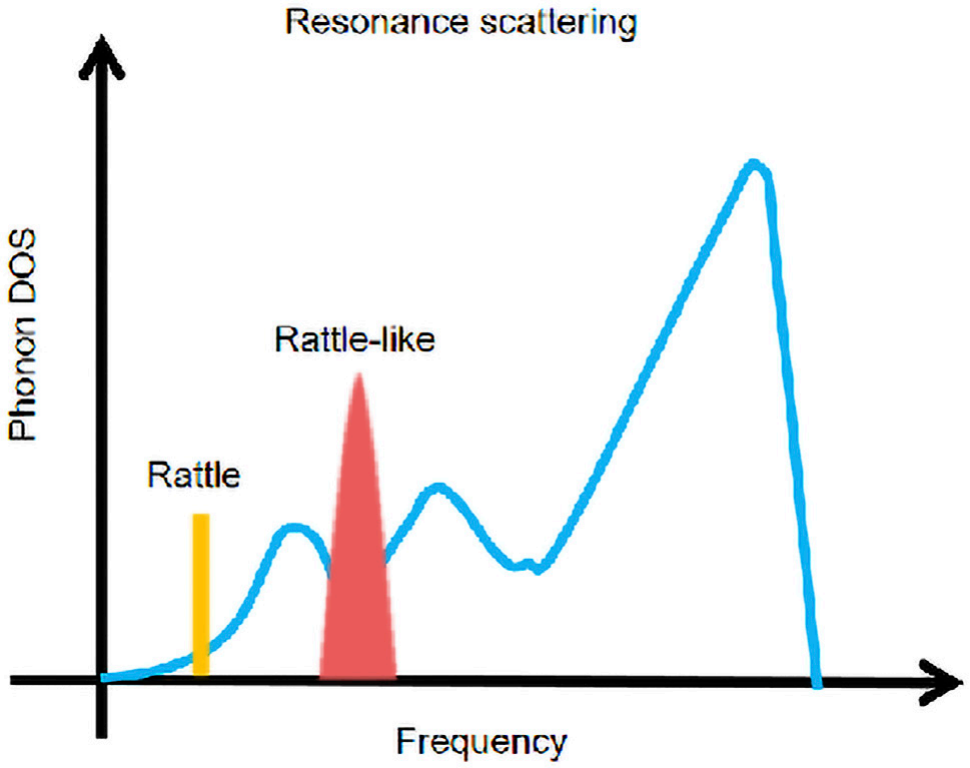
Schematic spectrum of resonance scattering at a specific frequency.

Resonance scattering is generally used in thermoelectric materials with special crystal structures, such as cage compounds (Christensen et al., 2008; Beekman and VanderGraaff, 2017), skutterudite (Duan et al., 2016), and some thermoelectric materials with topological insulation (Wei et al., 2016; Zeugner et al., 2019). By adding filler atoms, a strong local vibration close to the vibration frequency is generated, and a resonance spectrum of a specific frequency (shown in Figure 11) is introduced, thereby reducing the thermal conductivity.

**FIGURE 12:**
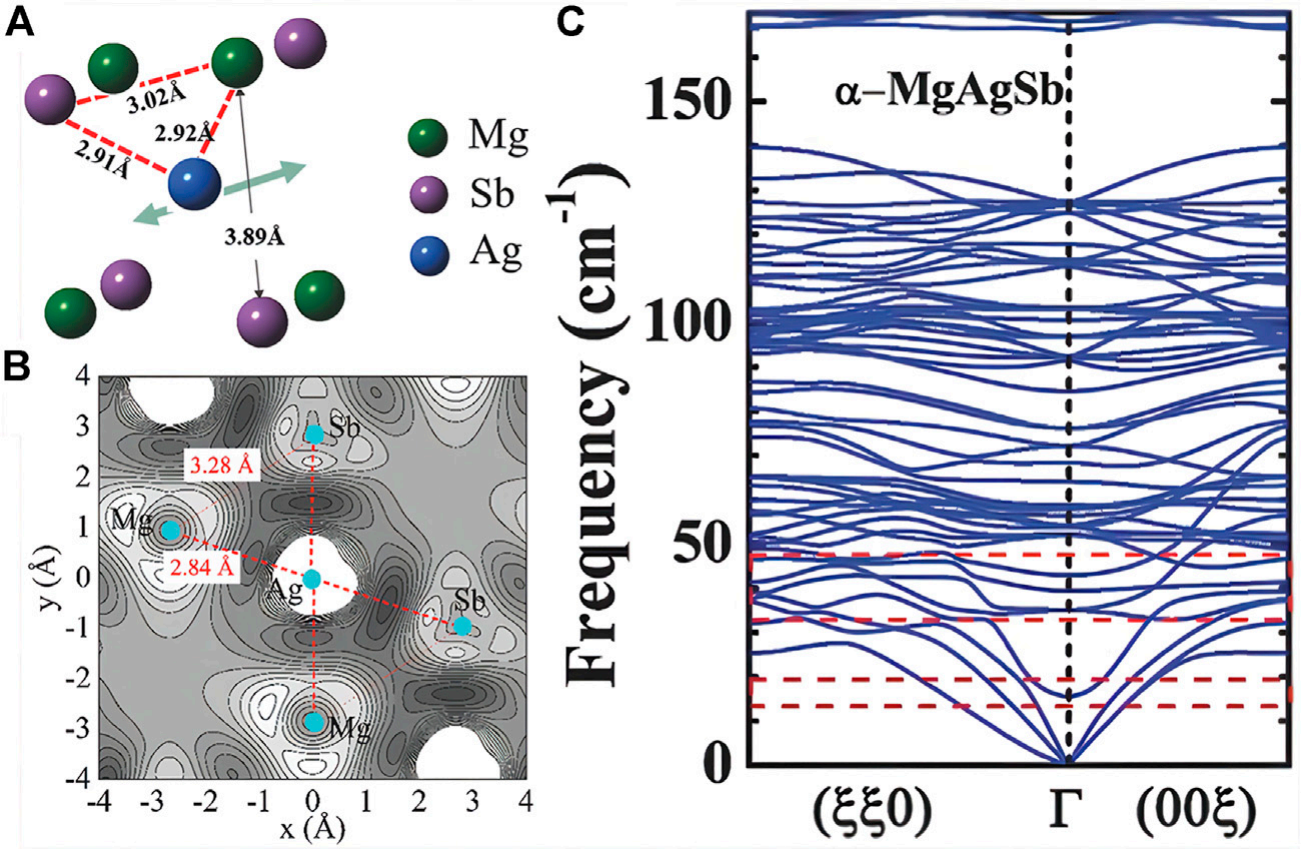
**(A)** Schematic diagram of the three-center bond structure in α-MgAgSb; **(B)** α-MgAgSb charge distribution diagram; **(C)** α-MgAgSb phonon spectrum (Ying et al., 2017).

Researchers fill CoSb_3_ with S and Se as electronegative objects under equilibrium conditions. Because S and Se have unique localized “cluster vibrations,” a section can be introduced in the phonon spectrum. The optical branch with lower frequency is coupled with the acoustic branch, which significantly affects the lattice dynamics, greatly reduces the thermal conductivity of the lattice, and obtains S_0.26_Co_4_Sb_11.11_Te_0.73_ with *ZT* = 1.5 at 850 K (Zeugner et al., 2019). In recent years, research on topological insulating thermoelectric materials has gradually increased, and many studies have also shown that some of them have resonance scattering. Samanta et al. (2018) reported a layered structure of n-type BiSe (Se-Bi-Se-Bi-Se), the study found that the local vibration generated by the Bi layer can cause some low-energy optical branches to inhibit the propagation of the acoustic branches (i.e., photoacoustic coupling), and the sound wave branch with low cut-off frequency and strong anharmonic vibration Phonon interaction further reduces the thermal conductivity, and finally *κ*_*L*_ = 0.3 W m^−1^ K^−1^ at 300 K. Similarly, Wei et al. (2016), Christensen et al. (2008) predicted the minimum thermal conductivity of bismuth-based stacked structures with topological insulation and found that the NI layer has a strong influence on Bi_2_TeI lattice dynamics and impedes phonon propagation, with low-frequency photo-phonon and phonon interactions arising from local vibrations of the NI layer causing a high degree of lattice anharmonicity and producing extremely low thermal conductivity. Moreover, Ying et al. (2017) studied the cause and physical mechanism of the intrinsic low thermal conductivity of α-MgAgSb thermoelectric materials and predicted a class of thermoelectric material systems with low thermal conductivity. The study found that α-MgAgSb has weak chemical bonds in addition to layering. In addition to the low sound velocity, the half-Heusler-like twisted lattice makes it form a Mg-Ag-Sb triple-center bond structure (Figures 12A,B). The Ag atoms in the Mg-Ag-Sb tri-center bond can move reciprocally in the direction of the arrow to a greater extent, forming a low-frequency optical branch in the phonon spectrum (Figure 12C). These low-frequency optical branches can generate resonance scattering and effectively reduce the thermal conductivity of the lattice (Ying et al., 2017). It can be seen from the above that the research on reducing thermal conductivity through resonance scattering is becoming more mature.

## Thermoelectric Materials With Low Thermal Conductivity

The use of low-dimensional defects and nanocomposite structures to reduce multi-scale phonon scattering and reduction of existing TE materials *к*_*L*_ does significantly increase the *ZT* value of thermoelectric materials, but existing methods at this stage will affect thermal stability and other issues. The search for thermoelectric materials with intrinsically low thermal conductivity is therefore also a necessary way to avoid the problems associated with reducing the thermal conductivity of existing materials. Most of the thermoelectric materials with an intrinsic low thermal conductivity that have been discovered so far have the characteristics of weak chemical bonding (Zhao et al., 2014a), strong anharmonic effects (Zhao et al., 2014d), liquid ionic properties (Liu et al., 2012), heavy molecular mass (Toberer et al., 2010), complex crystal structure (Kurosaki et al., 2005), etc. We will briefly describe the representative thermoelectric materials from the following five points.

### Weak Chemical Bond

The lattice thermal conductivity is proportional to the speed of sound, so obtaining low sound speed performance often helps to improve the thermoelectric properties of the material. Materials with weak chemical bonds have a lower speed of sound, atoms have more space for movement near their equilibrium positions, and electron cloud distribution is more diffuse. In the phonon spectrum, weak chemical bonds often correspond to some low-frequency phonon models, which are easier to couple with the acoustic branch, thereby further reducing the contribution of the acoustic branch to the thermal conductivity.

**FIGURE 13:**
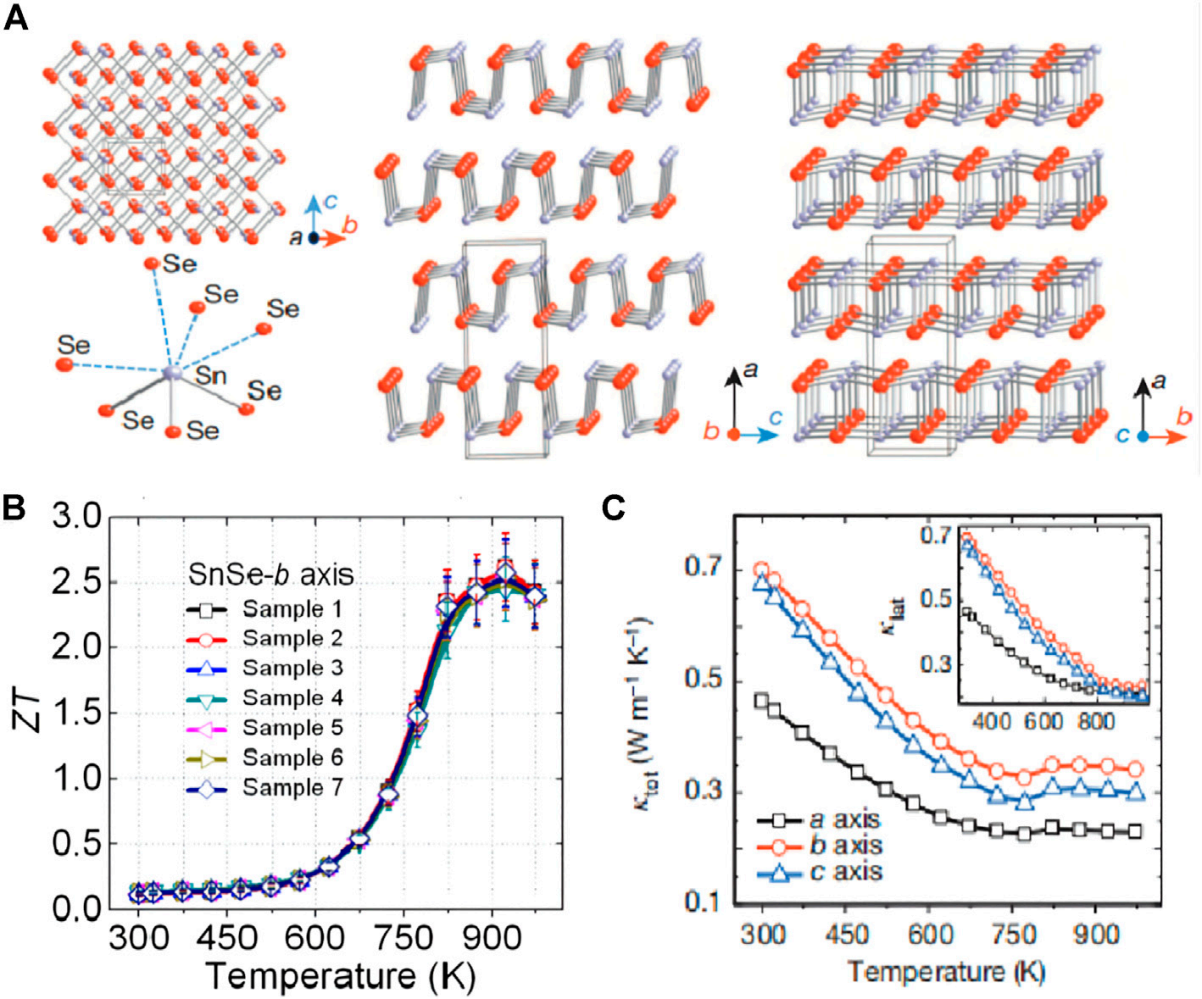
**(A)** SnSe crystal structure along the a-axis: gray, tin atoms; red, selenium atoms; highly distorted SnSe_7_ coordination polyhedron with three short and four long Sn-Se bonds; structure along the b-axis; Structure along the c axis; **(B)** SnSe along the b axis *ZT* value changes with temperature (T); **(C)** SnSe single crystal lattice thermal conductivity (*к*_*L*_) changes with temperature (T) in the a, b, and c directions (Zhao et al., 2014a).

SnSe is a typical simple thermoelectric material with weak chemical bonds and is very stable. SnSe has an orthogonal layer structure in the room temperature range. The Sn-Se bond in the bc direction is stronger and weaker in a direction. It is easy to cleave along the (100) plane and form a foldable projection in the b-axis direction. The structure contains a highly distorted SnSe_7_ coordination polyhedron with three short Sn-Se bonds and four long Sn-Se bonds. Such a special structure makes it have strong non-resonance and high anisotropy, which essentially has ultra-low thermal conductivity. Lidong Zhao et al. prepared SnSe single crystal by the Briman method and found that the special crystal structure of SnSe (as shown in Figure 13A) made SnSe have a very low *к*_*L*_, and the room temperature thermal conductivity of SnSe in the direction of the axis a was about 0.47 W m^−1^ K^−1^. With the increase of temperature, the thermal conductivity at 973 K decreased to about 0.23 W m^−1^ K^−1^ (as shown in Figures 13A, C), and a historic breakthrough was made at 923 K with a *ZT* of 2.6 along the b-axis (as shown in Figure 13B) (Zhao et al., 2014a). Subsequently, Zhao et al. (2014a) synthesized an n-type SnSe crystal with a two-dimensional layered structure with *ZT* up to 2.8 (Chang et al., 2018). Similarly, based on the low thermal conductivity of SnSe, Qin et al. (2020) introduced SnSe_2_ as an extrinsic defect dopant to increase the carrier concentration to 6.55 × 10^19^ cm^−3^ while increasing the effective Mass and Seebeck coefficient, obtained an unusually high power factor at room temperature (about 5.4 × 10^–2^ W m^−1^ K^−2^), so that the maximum *ZT* value reached 2.2 and the average *ZT* value reached 1.7.

In addition to single crystals SnSe, polycrystalline SnSe has gradually been widely used in research due to its thermal conductivity comparable to that of single crystals SnSe. Although SnSe crystals have strong anisotropy, polycrystalline materials have more carrier migration than single crystals SnSe materials. The rate is much lower, and the conductivity is very low, but therefore, increasing the conductivity has become an important way to improve the thermoelectric properties of polycrystalline SnSe. Zhang et al. (2015) prepared n-type SnSe polycrystalline powder by using I doping and SnS solid solution, and processed it in a vacuum device, and found that the thermal conductivity of polycrystalline SnSe would decrease, approaching 0.30 W m^−1^ K^−1^ at 773 K, which makes *ZT* = 1 finally.

Perhaps because polycrystalline materials have many dislocations, vacancies, bond deformations, and other defects in the grain boundary during the powder metallurgy process, they are in a state of stress distortion, and the energy is high, which makes the material in an unstable state at high temperatures, resulting in relatively poor performance. There are many speculations about the reason for the low thermal conductivity of SnSe. For example, the spring-type crystal structure of SnSe has a great shock-absorbing and buffering effect on the transmission of phonons in it (Heremans, 2014). Therefore, the thermal conductivity of SnSe still needs further investigation and verification.

### Non-Resonance Effect

The resonance effect refers to the balance of external forces experienced by phonons during the transmission process. The sound wave transmits uniformly forward in the form of a parabola, which is similar to the transmission in a perfect lattice, but when the transmission of phonons is affected by external factors, it will deviate from the equilibrium position and cause non-resonance effects. The strength of anharmonicity is mainly related to the symmetry of chemical bonds and atomic equilibrium positions. During the vibration of an atom, the greater the deviation of its symmetry center, the stronger the asymmetry. Materials with lone pairs of electrons are often due to the uneven distribution of the electron cloud, the crystal structure will be deformed to a certain extent, and the asymmetry is significantly enhanced, which is beneficial to obtain strong anharmonicity.

Because there are two isolated electrons in the outermost layer of the Sb nucleus. The two lone electrons in the outermost layer of the nucleus will make the transmission of phonons unbalanced, resulting in non-resonance effects, resulting in Sb compounds with low heat transport properties. In the same way, compounds with Bi atoms belonging to the same group as Sb atoms should also have anharmonic effects. Therefore, the common thermoelectric materials with intrinsic low thermal conductivity of this kind are AgSbSe_2_, BiCuSeO, and so on. The room temperature thermal conductivity of AgSbSe_2_ is 0.48 W m^−1^ K^−1^ (Nielsen et al., 2013), which is lower than most thermoelectric materials at room temperature. In recent years, some people have introduced AgSbSe_2_ into GeTe to prepare (GeTe)_80_ (AgSbSe_2_)_20_. The introduction of hierarchical nano/mesoporous structure inside the material causes large-scale scattering of phonons, which reduces the lattice thermal conductivity *к*_*L*_ to 0.4 W m^−1^ K^−1^ at 300–700 K, which is close to the thermal conductivity of GeTe. The theoretical limit of the rate finally makes its *ZT*_*max*_ reach 1.9 at 660 K (Samanta et al., 2017). Later, Gao et al. (2018) used the method of mechanical alloying and rapid hot pressing to manufacture Ca doped AgSbSe_2_ with the layered structure. Ca doping further optimized the carrier concentration and caused the point defect scattering of phonons, and obtained the ultra-low lattice thermal conductivity of 0.27 W m^−1^ K^−1^ at 673 K, thus increasing the *ZT* peak value to 1.2.

Since BiCuSeO was reported in 2010 (Zhao et al., 2010), BiCuSeO is considered to be one of the most promising oxygen-containing thermoelectric materials due to its special natural superlattice structure and corresponding ultra-low thermal conductivity. Later, researchers found that in BiCuSeO, modulating doping to increase carrier mobility can greatly improve thermoelectric performance. Modulating the heterostructure of the doped sample makes the carriers preferentially transport in the low carrier concentration region, which increases the carrier mobility by 2 times while maintaining the carrier concentration similar to the uniformly doped sample. The synergistic effect of the increased conductivity and the basically constant Seebeck coefficient results in a wide range of high power factors, about 5–10 × 10^–2^ W cm^−1^ K^−2^. The combination of ultra-high power factor and extremely low thermal conductivity (about 0.25 W m^−1^ K^−1^) enables BiCuSeO to obtain a high *ZT* ≈ 1.4 at 923 K (Pei et al., 2014)^**.**^

Later, with the widespread acceptance of simulation calculations, Saha (2015) used first-principles density functional theory calculations to propose the atomic displacement map of the lowest frequency optical mode of BiCuSeO, and systematically studied the correlation of BiCuSeO (such as dielectric and Anharmonic) characteristics, and compared with LaCuSeO of the same structure, it is found that the reason for the ultra-low thermal conductivity *κ* of BiCuSeO is that the Bi atom exhibits a significant displacement during the action, which indicates that it has a higher anharmonic effect. Furthermore, the calculated out-of-plane thermal conductivity of BiCuSeO is approximately twice that of the in-plane thermal conductivity, which indicates that BiCuSeO has a greater anisotropy of heat flow between in-plane and out-of-plane.

### Ionic Liquid Characteristics

In solid, *C*_*v*_ = 3*N*_*k*_ *B,* while in liquid, most transverse vibration waves cannot be transmitted, *C*_*v*_ decreases to 2–2.5*N*_*k*_ *B*, and *N*_*k*_ is the total atomic number. Therefore, materials with liquid properties often have relatively low thermal conductivity due to their small heat capacity. Ionic conductors introduce some ions with “liquid” characteristics into solid materials to form an ionic liquid-like semiconductor, thus reducing the lattice thermal conductivity to a level lower than that of glass, which can not only reduce the mean free path of phonons but also eliminate lattice vibration. Cu_2−x_Se compounds belong to this class of ion-electron composite conductors with special structures with two-phase structures at room temperature (transition from low-temperature *α* phase to high-temperature *β* phase at 400 K). The lattice network structure of Cu_2−x_Se face-centred cubic Se atoms provides a good electrical transport pathway for the randomly distributed free movement of Cu ions in the interstitial positions, which enhances the scattered lattice phonons while reducing some lattice vibrational modes, so that the lattice heat capacity of the material decreases with increasing temperature and ultimately low thermal conductivity properties can be obtained.

Skomorokhov et al. (2006) experimentally found the local migration of Cu atoms in Cu_2_Se. Later, Liu et al. (2012) found that the abnormal “liquid-like” behavior of copper ions around the crystalline sublattice of Se in Cu_2−x_Se (Figure 3 shows the simple Face-centered cubic structure of Se atoms in the High-temperature *β* phase) leads to essentially very low lattice thermal conductivity (0.4–0.6 W m^−1^ K^−1^), thus achieving high *ZT* = 1.5 (*T* = 1,000 K) in this originally simple semiconductor. This unusual combination of properties resulted in ideal thermoelectric materials. The results show that exploring the network structure with electron conduction sublattices surrounded by liquid ions can provide new strategies and directions for High-efficiency thermoelectric materials.

Subsequently, Zhong et al. (2014) generated highly aligned wafers in the bulk Cu_1.94_Al_0.02_Se by the direct current hot pressing process according to the alternating arrangement of single disordered layers of Se ions and double disordered layers of Cu ions. The superionic mechanism in Cu_1.94_Al_0.02_Se was further enhanced by the enhancement of Cu ions and high crystal orientation. Finally, *ZT* = 2.62 was obtained at 756°C (Zhong et al., 2014). This study also provides a reliable way to use the superior mechanism. In the research process of Cu_2−x_Se, the high fluidity of copper ions causes problems such as thermal stability during the preparation of Cu_2−x_Se, which prevents the development of Cu_2−X_Se. Therefore, it is also necessary to consider how to control such problems in the research process.

### Molecular Weight

Heat capacity *C*_*v*_ is the important factor influencing the thermal conductivity and is proportional to the lattice thermal conductivity, based on the debye model for the heat capacity, the acoustic wave is the main source of contribution to the thermal conductivity, the assumption in a polyatomic system, the total number of the original cell of N, every cell in the atomic number is n, total system degrees of freedom to 3 nN, among them, the acoustic branch has 3N, optical 3(n−1)N, by the equipartition principle, the contribution of the acoustic branch heat capacity as the C/n, optical to C(n−1)/n (Toberer et al., 2011). Therefore, the total heat capacity remains unchanged. The more atoms in the primary cell, the less contribution of acoustic branches to the total heat capacity is, and the more conducive to obtaining low lattice thermal conductivity, which means that the thermoelectric materials with heavy molecular weight have certain advantages to obtain excellent thermoelectric properties. This has been demonstrated in many thermoelectric materials with complex crystal structures.

**FIGURE 14:**
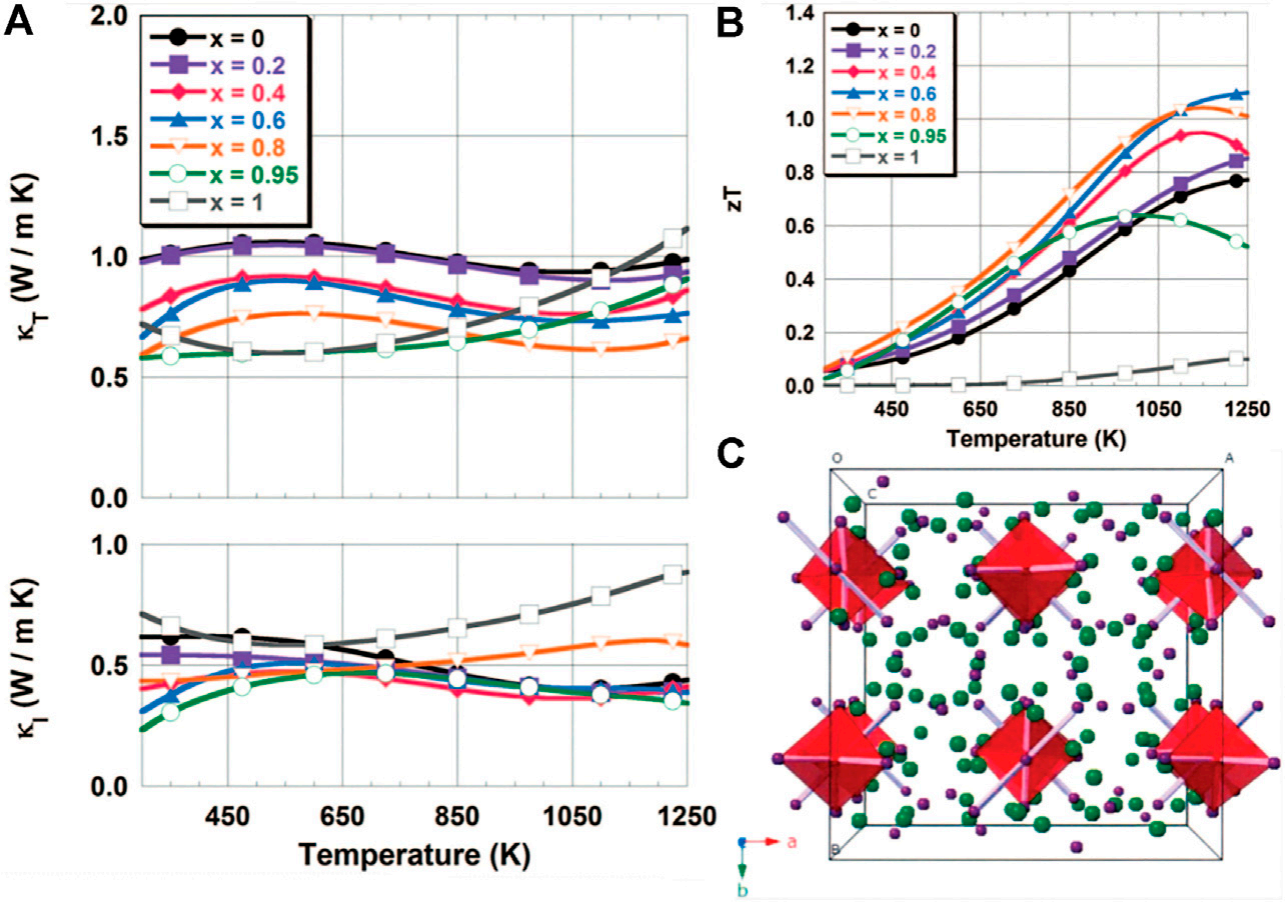
**(A)** Variation of total thermal conductivity and lattice thermal conductivity of Yb_14_Mn_1-x_Al_x_Sb_11_ with *x*; **(B)** The change of *ZT* with the temperature at different *x*; **(C)** The crystal structure of Yb_14_Mn_1-x_Al_x_Sb_11_ (Cox et al., 2009).

Zintl phase Yb_14_MnSb_11_ is a typical macromolecular mass material with a relative molecular weight of 3,783.088 g/mol, and its crystal structure is shown in Figure 14C (Wang Y. et al., 2018). Brown et al. (2006) found that electronic and thermal properties could be potentially modulated by doping at different locations in the A_14_MPn_11_ class of structures. Doped at cationic metal site A, the carrier concentration and phonon disordered scattering can be adjusted. Doped at cationic metal site A, the carrier concentration and phonon disordered scattering can be adjusted. Doping at a metal site M can adjust the electronic parameters, and for the first time by preparing Zintl phase Yb_14_MnSb_11_ to obtain the thermoelectric performance of the stability at high temperature, the thermal conductivity in about 0.7–0.9 W m^−1^ K^−1^ (400–1,300 K), the Seebeck coefficient reaches the maximum value of 185 μV/K at 1,275 K, and the *ZT* value reaches one at 1,223 K, which provides new guidance for the study of Zintl phase Yb_14_MnSb_11_.

Then, Cox et al. (2009) began to conduct Al doping on the metal sites of Yb_14_Mn_1−x_Al_x_Sb_11_ structure to regulate electronic parameters. Studies have shown that within a certain range of heat capacity (300–1,100 K), as the aluminum content increases, the unit cell volume increases, and the bond distance changes less than 2%, which means that the increase in cell volume is related to the decrease in the deformation of the tetrahedron with the increase in Al content. When the *ZT* peak appears at 1,223 K, it deviates from the measurement range, indicating that the lattice thermal conductivity here is extremely low (about 0.3–0.4 W m^−1^ K^−1^), as shown in Figure 14A. Subsequently, Uvarov et al. (2012) improved Seebeck’s coefficient and reduced thermal conductivity by replacing Yb^2+^ with equivalent Ca^2+^ in Yb_14_MnSb_11_, thus increasing *ZT* and obtaining higher thermoelectric performance. Therefore, it can be seen that the thermoelectric materials with similar heavy molecular weight can be adjusted at different sites to obtain low lattice thermal conductivity and stable thermoelectric properties with great exploration value.

### Complex Crystal Structure

It is well known that the main factors affecting the thermal conductivity of materials are heat capacity, sound velocity, and phonon relaxation time. The complex crystal structure not only reduces the contribution of the acoustic branches to the total heat capacity, but also reduces the group velocity of the acoustic branches.

**FIGURE 15:**
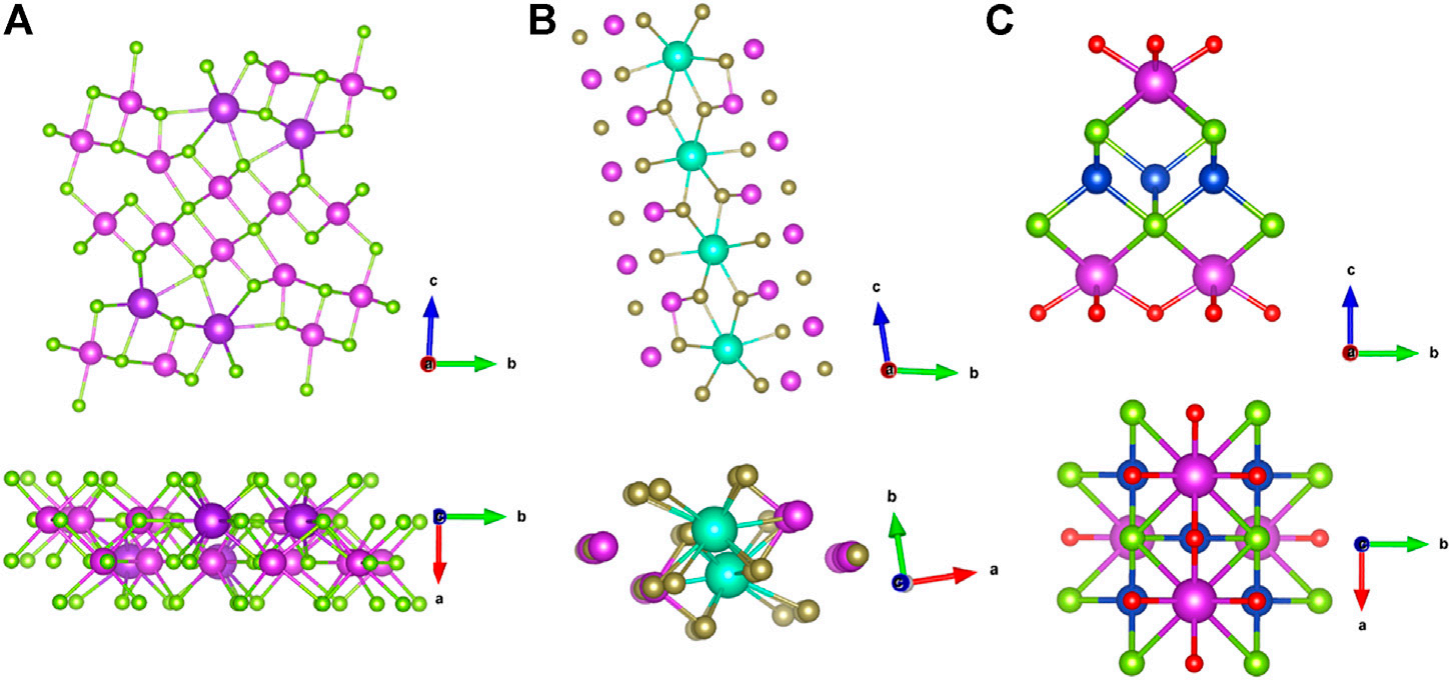
Crystal structure of complex thermoelectric materials: **(A)** Crystal structure of β-K_2_Bi_8_Se_13_; **(B)** Crystal structure of CsBi_4_Te_6_; **(C)** Crystal structure of BiCuSeO.

Due to its complex crystal structure, Ag_9_TlTe_5_ has a heavy molecular mass, and the number of atoms in a single cell is 298. Meanwhile, the low elastic modulus indicates that the chemical bond of the material is weak. The thermal conductivity of Ag_9_TlTe_5_ remains about 0.25 W m^−1^ K^−1^ from room temperature to 650 K (Kurosaki et al., 2005), which makes the *ZT* of the material reach 1.25 at 673 K. The ternary alkali metal-bismuth-chalcogenides exhibit extremely low thermal conductivity due to their more complex crystal structure, such as β-K_2_Bi_8_Se_13_ (Figure 15A), CsBi_4_Te_6_ (Figure 15B), etc. The low symmetry monoclinic crystal structure of β-K_2_Bi_8_Se_13_ makes it highly anisotropic. β-K_2_Bi_8_Se_13_ was first proposed by Meng et al. (2003), and its thermal conductivity is as low as 1.28 W m^−1^ K^−1^. Afterwards, Kyratsi et al. (2011)prepared crystalline/amorphous β-K_2_Bi_8_Se_13_ nanocomposites by ball milling the melt-synthesized polycrystalline β-K_2_Bi_8_Se_13_ under an inert atmosphere. It was found that with the time of ball milling, the nanocrystalline phase gradually formed, the carrier concentration gradually decreased, the Seebeck coefficient gradually increased, and the thermal conductivity gradually decreased from the initial 1.28–0.3 W m^−1^ K^−1^, which greatly improved the thermoelectric properties of β-K_2_Bi_8_Se_13_, and provided a new and reliable idea for the follow-up research. In addition, there are many materials with complex crystal structures, such as Bicuseo (Figure 15C) and Yb_14_Mn_1-x_Al_x_Sb_11_ mentioned above, all of which are more or less affected by their structures. Therefore, given the many materials yet to be investigated, there is certainly much work ahead and promise for developing TE materials.

## Electron-Phonon Decoupling

In the above, we discussed that nanostructures in bulk materials greatly reduce thermal conductivity. However, due to the crystal mismatch or the electronic band of interface caused by the mismatch increased energy barrier, also can increase the carrier scattering, the carrier mobility and power factor have a negative effect, in other words, when doping material scale and the rise in the Numbers, thermal conductivity and electrical conductivity of coherence will increase, not conducive to improve the performance of thermoelectric (Zhao et al., 2013a). Therefore, ensuring high carrier mobility and wideband gap while fully regulating the thermal conductivity has become the key to the breakthrough of thermoelectric materials. The decoupling mode of electron and phonon transport is discussed below.

Slack proposed the concept of “Phonon glass and Electron Crystal” (PGEC), which can be used to guide the optimal design of thermoelectric materials. He believed that an ideal thermoelectric material should have phonon transport characteristics like glass and electron transport characteristics like crystal (Slack and Rowe, 1995). At present, the introduction of nano-sized precipitates can increase phonon scattering and produce very little electron scattering through interfacial interaction. When there is a coherent connection between the nano-precipitated phase and the matrix, but there is a certain tension around the interface (with nanostructure inside the tension), this interface is considered to promote the charge passing through, but strongly hinders the propagation of scattered phonons (Hsu et al., 2004; Cook et al., 2009). The coherent strain interface between the substrate and the nanometer precipitates results in strong phonon scattering, but the electron scattering is very small. Unfortunately, interfacial coherence can sometimes lead to asymmetry of energy band structure and generate potential barriers to hinder electron migration (Biswas et al., 2011). Although electron scattering can be minimized by improving the interfacial coherence of nanostructured materials, the energy barrier created by the asymmetry of electron bands hinders electron flow. Therefore, atomic fixation or nanometer phase minimization is generally used to increase electron migration. Huang et al. (2020) synthesized CuGaTe_2_ + x wt.% CPS(*x* = 0, 0.3, 0.5, 1) composites. The results show that carbon particles (CPS) can be introduced into CuGaTe_2_ matrix as electronic location center, and the conductivity and power factor of CuGaTe_2_ matrix can be improved by adjusting its electrical transport performance. The results show that the introduction of CPS can be used as the local electron center, thus the hole concentration in the matrix is increased, and the electrical conductivity is increased by about 120% (300 K). Besides, the interface between carbon particles and matrix enhances the scattering of heat-carrying phonons and reduces the thermal conductivity of CuGaTe_2_ + 0.5 wt.% CPS sample at 873 K, which significantly reduces the thermal conductivity of CuGaTe_2_ sample by 37% compared with pure CuGaTe_2_ (Huang et al., 2020).

To ensure the micro-transfer of energy bands and even form band alignment structure when embedded in nano-scale, the design of alloyed nano thermoelectric materials is also a good approach (Zhao et al., 2013b). The double alloying method of PbTe adopted by Sumanta Sarkar combines two different but identical structures, which can effectively improve the thermoelectric properties of PbTe from two aspects. First, in the presence of Bate, the solubility of Cate increases, leading to a widening of the band gap and a good convergence between L and ∑ bands, resulting in a record-high PF for the Seebeck coefficient. In addition, the point defects generated by alloying and the BaT-rich nanostructures contribute to the reduction of the lattice thermal conductivity of the system. This synergistic method generates a high *ZT* of 2.2 at 823 K (Sarkar et al., 2018). Makongo et al. (2011) proposed to obtain half-Heusler and Heusler nanocomposite phases *in situ* by atomic-scale regulation, which effectively improved the electrical conductivity and power factor of the material and reduced the lattice thermal conductivity of the material. At the same time, Jie Ma et al. studied the fundamental electron-phonon interaction using the chemical doping of ZrNis-based semi-Hessler materials as an example (Ren et al., 2020). This crossover between different major scattering mechanisms is achieved by shielding ionized impurities, grain boundary, and polarized phonon scattering, and enhanced phonon/alloy scattering. Based on the understanding of different carrier scattering, they derived the carrier scattering phase diagram, which can guide further improving the Te properties of semi-Hessler Te materials. This method can also be extended analogically to materials of different systems and may provide excellent guidance for electro-acoustic decoupling.

Organic materials would be a better choice purely from the direction of reducing thermal conductivity. However, the biggest disadvantage of organic materials is that their electrical properties are difficult to regulate, which greatly affects their thermoelectric properties. The thermal conductivity of organic thermoelectric materials is usually lower than 1 W m^−1^ K^−1^, close to the lower limit of the thermal conductivity of inorganic thermoelectric materials (Huang et al., 2016). Moreover, the electronic structure of the organic (semi) conductor can be appropriately adjusted by molecular chemistry and doping treatment, so that electron transport is not affected (Ren et al., 2018). Wang L. et al. (2018) not only reduced the thermal conductivity through flexible organic-inorganic hybridizationbut also improved the electrical performance, effectively realizing electro-acoustic decoupling. Therefore, organic thermoelectric materials have great prospects in Low-temperature applications.

The above is mainly by suppressing phonon transmission to ensure electron transport to improve the thermoelectric performance. The difference is that Zhimei Sun et al. discussed the thermoelectric properties of two-dimensional Nb_2_C through first-principles calculations. It is found that when the density of electron states near the Fermi level is high and the phonon frequency is high, the anomalous strong E-P scattering will be generated, and the thermal conductivity of the lattice will be greatly reduced, which also provides a new idea for the design of two-dimensional metallic thermoelectric materials (Huang et al., 2019).

## Conclusion

Although thermoelectric functional materials have a long history of research, from the discovery of narrow-band gap semiconductor materials to the vigorous development of wideband gap semiconductor thermoelectric materials, they still fail to achieve excellent ideal performance, so there is still a lot of room for exploration and improvement. This paper elaborates on the improvement of the thermoelectric optimal value *ZT* from the aspects of optimizing carrier concentration, improving carrier mobility, increasing effective mass, reducing lattice thermal conductivity, exploring intrinsic thermoelectric materials, and electro-acoustic decoupling, among which the relatively independent lattice thermal conductivity regulation has received extensive attention.

In energy band engineering, we can improve the effective mass by introducing resonant state density, increasing energy band degeneracy, controllable hybridization gap and doping Kondo atoms. However, increasing the Seebeck coefficient by increasing the effective mass tends to reduce carrier mobility. At the same time, the Seebeck coefficient can be improved by optimizing the carrier concentration and increasing carrier mobility by modulating doping and temperature-dependent gradient doping. In phonon engineering, the mechanism of point defect scattering, dislocation scattering, interface scattering and resonance scattering to reduce lattice thermal conductivity is summarized. It also reveals the structure-activity relationship of several influencing factors such as weak chemical bonding, strong anharmonic effects, liquid ionic properties, heavy molecular mass and complex crystal structure on the intrinsic low thermal conductivity thermoelectric materials. Finally, the electro-acoustic decoupling theory, such as how to block the transmission of electrons by phonons, is briefly explained.

It can be seen that further exploration of electro-acoustic synergy mechanism and design of intrinsic low thermal conductivity is still the mainstream research direction, but when making some breakthroughs, it is faced with challenges such as thermal dynamic stability and other issues. We can start with the following strategies: 1) new mechanism, new concept, and new theory of the synergy between band engineering and phonon engineering; 2) the influence mechanism of material defect engineering on thermoelectric properties; 3) the influence of synthesis methods, and characterization methods on the evolution of interface, and microstructure; 4) To design a comprehensive theoretical calculation module and establish a complete material genome database to screen new thermoelectric materials with high performance at a high speed and effectively. Although the influence conditions of thermoelectric performance are interlaced and complex, it is believed that breakthrough progress will be made under the rapid development of materials, physics, chemistry and computer science, and it is bound to play a role in the future reliable clean energy.
